# Unraveling the Pathogenetic Potential and Immune Crosstalk of *Yersinia* spp. in Inflammatory Bowel Disease

**DOI:** 10.1155/jimr/2846665

**Published:** 2026-08-28

**Authors:** Fattaneh Sabzehali, Verena Zerbato, Stefano Di Bella, Stefano Morabito, Mohammad Reza Zali, Abbas Yadegar

**Affiliations:** ^1^ Foodborne and Waterborne Diseases Research Center, Research Institute for Gastroenterology and Liver Diseases, Shahid Beheshti University of Medical Sciences, Tehran, Iran, sbmu.ac.ir; ^2^ Infectious Diseases Unit, Trieste University Hospital (ASUGI), Trieste, Italy; ^3^ Clinical Department of Medical, Surgical, and Health Sciences, Trieste University, Trieste, Italy, units.it; ^4^ Department of Food Safety, Nutrition and Veterinary Public Health, Istituto Superiore di Sanità, Viale Regina Elena, 299, Roma 00161, Italy, iss.it; ^5^ Gastroenterology and Liver Diseases Research Center, Research Institute for Gastroenterology and Liver Diseases, Shahid Beheshti University of Medical Sciences, Tehran, Iran, sbmu.ac.ir

**Keywords:** Crohn’s disease, host–pathogen interaction, inflammatory bowel disease, innate immunity, ulcerative colitis, *Yersinia enterocolitica*, *Yersinia pseudotuberculosis*

## Abstract

*Yersinia enterocolitica* and *Yersinia pseudotuberculosis* have been identified as microbiological factors in the multifactorial pathophysiology of inflammatory bowel disease (IBD). Although conventionally associated with self‐limiting gastroenteritis, *Yersinia* has been increasingly investigated for its potential role in IBD, particularly in genetically predisposed individuals. Whether this reflects causality, opportunistic colonization, or secondary enrichment within inflamed tissue, however, remains unresolved. Polymerase chain reaction (PCR)‐based studies have detected *Yersinia* DNA in the intestinal tissues of patients with IBD, and epidemiological studies have suggested an association between prior *Yersinia* infection and an increased long‐term risk of disease. However, because most evidence is based on PCR detection, with limited culture‐based confirmation of viable organisms and infrequent sequencing of PCR amplicons, these findings do not establish active infection or a causal relationship. These pathogens exploit abnormalities in the epithelial barrier and compromised mucosal immunity through type III/VI secretion systems to disrupt host responses, activate inflammasomes, and induce pyroptosis. Polymorphisms in autophagy‐related genes, such as NOD2 and ATG16L1, impair pathogen clearance and increase susceptibility. While antibiotics continue to be effective against invasive diseases, the rise of resistance highlights the necessity for alternatives. Probiotic‐based strategies have demonstrated immunomodulatory, antimicrobial, and barrier‐protective effects in preclinical models, although their therapeutic efficacy in IBD requires further clinical validation. Comprehending *Yersinia*–host genetic interactions could facilitate precision diagnostics and microbiota‐targeted treatments in IBD.

## 1. Introduction

Crohn’s disease (CD) and ulcerative colitis (UC) are two principal forms of inflammatory bowel disease (IBD) defined by chronic, severe, and progressive conditions that result in recurrent gastrointestinal inflammation [1]. CD can affect any part of the digestive tract, commonly impacting the ileum and proximal colon, and is characterized by significant inflammation. Conversely, UC mainly impacts the colon and rectum, typically presenting as superficial inflammation limited to the mucosal layer of the colon [2]. Although global age‐standardized rates of prevalence, mortality, and disability‐adjusted life‐years (DALYs) for IBD declined modestly from 1990 to 2019, the absolute number of affected individuals increased in 147 of the 204 countries and territories examined, underscoring the continuing rise in the global burden of IBD [3]. In 2019, IBD was estimated to affect more than 4.9 million individuals worldwide. Traditionally seen as a “Western disease,” this viewpoint is evolving as incidence rates stabilize in Western nations while increasing in parts of Asia, South America, and the Middle East [4]. The etiology of IBD remains incompletely elucidated, though it is thought to result from a complicated interaction of genetic, environmental factors (such as physiological stress, diet, and smoking), immunological abnormalities, and microbial influences [5, 6].

Microbial imbalances can trigger an abnormal immune response, leading to inflammation of the gastrointestinal system [7]. Numerous studies have demonstrated that the composition of enteric microorganisms is altered in individuals with IBD, characterized by an excess of specific bacteria, such as *Bacteroides*, *Escherichia coli* (*E. coli*), and *Enterococcus*, alongside a reduction in beneficial bacteria, including *Prevotella* and *Akkermansia* [8–10]. Research indicates that imbalances in microbial families, including Rikenellaceae, Christensenellaceae, Veillonellaceae, Bacteroidaceae, and Ruminococcaceae, contribute to the worsening of IBD [11–15]. Recent advances have expanded the concept of dysbiosis beyond broad taxonomic shifts to include disease‐associated pathobionts, such as adherent‐invasive *E. coli* (AIEC) and *Ruminococcus gnavus*, which contribute to epithelial barrier disruption, persistent immune activation, and chronic intestinal inflammation [16, 17]. Moreover, alterations in bacteriophage communities, fungal dysbiosis, and mucosal‐associated microbiota have emerged as important modulators of microbial ecology and host immune responses in IBD [18–20].

Among the enteric pathogens associated with IBD, *Yersinia enterocolitica* and *Yersinia pseudotuberculosis* deserve particular attention because they provide a unique model for understanding the interaction between microbial infection and host genetic factors in CD. Experimental and clinical studies have demonstrated that these pathogens interact with key NOD2‐ and ATG16L1‐dependent pathways, preferentially target the terminal ileum and mesenteric lymph nodes, and exploit defects in epithelial barrier integrity, autophagy, and innate immune responses [21–24]. Consequently, *Yersinia* has become an important model for elucidating host–pathogen interactions and the molecular mechanisms underlying CD pathogenesis. These zoonotic bacteria are transmitted primarily through contaminated food or water, are substantial contributors to worldwide foodborne disease [25]. *Y. enterocolitica*, the fourth most prevalent foodborne zoonosis in 2023 [26], exhibits significant clinical and pathological parallels with CD, which can lead to misdiagnosis and hinder patients from receiving timely and effective treatment [27]. Although *Y. enterocolitica* is commonly found in CD lesions, its exact involvement in the etiology of CD—whether as a passive observer or an active contributor—has yet to be clarified [28]. The ability of *Yersinia* infections to provoke intestinal inflammation, coupled with their detection in the intestinal tissues of patients with CD, suggests a possible role in disease development [29]. However, current evidence is insufficient to distinguish whether these observations reflect transient enteric infection, persistent colonization, or a direct contribution to chronic intestinal inflammation [21, 30].

This review explores the historical development, taxonomy, and bioserotype classification of *Y. enterocolitica* and *Y. pseudotuberculosis*, highlighting their ecological traits, host diversity, and zoonotic transmission pathways. This study further investigates the interaction between these pathogens and intestinal immunity, emphasizing their ability to exploit epithelial barrier breakdown and modify innate and adaptive responses relevant to CD and UC. Particular focus is given on host genetic susceptibilities—such as polymorphisms in NOD2 and ATG16L1—that influence the immunological consequences of *Yersinia*–host interactions in IBD. Furthermore, we assess existing therapeutic techniques, including antibiotic regimens and microbiome‐modifying methods, and examine how these interventions may reduce *Yersinia*‐related inflammatory processes in the gastrointestinal tract. This integrative strategy aims to reclassify *Yersinia* from random enteric pathogens into potential contributors to the complex and multifaceted nature of IBD pathogenesis.

## 2. History, Taxonomy and Bioserotypes Determination

**Figure 1:**
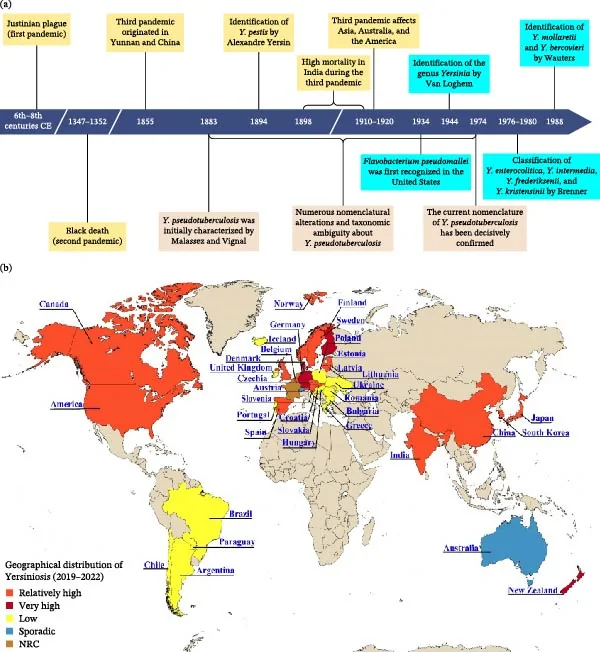
Chronological overview of key discoveries related to the genus *Yersinia* (a) and geographic distribution of yersiniosis per 100,000 populations (b). *Source:* European Centre for Disease Prevention and Control, yersiniosis, in ECDC, Foodborne Diseases Active Surveillance Network (FoodNet) in the United States, and www.myhealthunit.ca (2019–2022). CE, Common era. The figure was represented using https://www.mapchart.net/world-advanced.html.

The genus *Yersinia* belongs to the family Enterobacteriaceae and comprises 28 species [31], of which *Y. pestis*, *Y. enterocolitica*, and *Y. pseudotuberculosis* are pathogenic to humans. *Y. pestis* is classified as a Category A priority disease via the National Institute of Allergy and Infectious Diseases (NIAID) because of its potential for bioterrorism abuse and its past involvement in three significant pandemics [32]. The historical development of the *Yersinia* taxonomy is summarized in Figure 1a.

*Y. enterocolitica*, a heterogeneous species, and the more homogenous *Y. pseudotuberculosis* are often classified using biotyping (based on biochemical activity) and serotyping (based on lipopolysaccharide [LPS] O‐antigens) [33, 34]. Based on metabolic activity, *Y. enterocolitica* is classified into six biotypes. Highly virulent strains associated with human infection are primarily classified within biotypes 1B, 2, 3, 4, and 5 [35]. Among 70 distinct serotypes, O:3 (biogroup 4), O:8 (biogroup 1B, which causes a more severe disease), O:9 (biogroup 2), and O:5 (especially 5,27, biogroups 2 and 3) often infect humans [36, 37]. Researchers widely acknowledge that bioserotype 1B/O:8 is the most highly pathogenic, responsible for four out of the six food poisoning epidemics in the United States [38]. Studies have identified serotypes 4/O:3, 2/O:9, and 2/O:5.27 as the main agents causing human cases of yersiniosis in Europe [39–41]. In 2021, the European Food Safety Authority recorded 25 outbreaks of yersiniosis, with Denmark and Finland reporting the highest notification rates (Figure 1b) [42]. Annually, yersiniosis occurs at an average rate of 7.2 cases per 100,000 people in Germany, with children under five experiencing the highest incidence. Approximately 90% of infections originated from domestic sources, and bioserotype O:3 emerged as the predominant serotype [43]. Although the global burden of IBD has increased substantially over recent decades, particularly in newly industrialized regions, whether this trend parallels the epidemiology of yersiniosis remains unclear. These observations suggest that *Yersinia* is more likely to represent one of several environmental contributors to IBD rather than a primary determinant of its global epidemiological patterns.

## 3. Ecological Characteristics, Host Diversity, and Transmission Pathways

**Figure 2:**
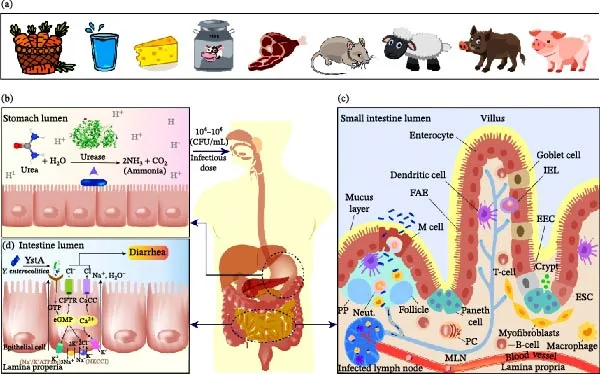
Overview of the ecological distribution, infection pathways, and pathophysiological mechanisms of human pathogenic *Yersinia* species, encompassing the action mechanism of *Yersinia*‐stable toxins (YST). (a) Enteropathogenic *Y. enterocolitica* and *Y. pseudotuberculosis* are associated with meat (mainly pork), wild boar, rodents, sheep, dairy, vegetables (lettuce, carrots), and contaminated water. (b) To survive gastric acidity, both species produce urease, which neutralizes low pH by generating ammonia. (c) After ingestion, both pathogens are phagocytosed by submucosal macrophages and phagocytic cells via phagosomes, then reach the lymphatic system through M cells in PP, allowing colonization and proliferation in the MLN. (d) YstA, an enterotoxin from *Y. enterocolitica* (biotypes 1B and 2–5), induces diarrhea by activating guanylate cyclase in epithelial cells, raising cGMP levels and promoting colonic fluid secretion. The YstA mechanism is known, but its receptor remains unidentified. CaCC, calcium‐activated chloride channels; CFTR, cystic fibrosis transmembrane conductance regulator; cGMP, cyclic guanosine monophosphate; EEC, entero‐endocrine cell; ESC, epithelial stem cell; FAE, follicle‐associated epithelial cell; IEL, intraepithelial lymphocytes; MLN, mesenteric lymph nodes; Neut., neutrophil; NKCC1, Na‐K‐2Cl cotransporter 1; PC, plasma cell; PP, Peyer’s patch.

Enteropathogenic *Yersinia* species are facultatively anaerobic, non‐spore‐forming bacilli responsible for gastrointestinal diseases in humans [44]. These organisms are predominantly spread via contaminated food or water, frequently leading to clinical manifestations such as diarrhea, stomach discomfort, and fever [45]. *Y. enterocolitica* is widely found in the environment and among animals, with pigs as the principal reservoir, in addition to other domestic and wild species that may enable zoonotic transmission [46]. *Y. pseudotuberculosis* was initially linked to a significant outbreak in 1998 related to contaminated iceberg lettuce [47]. Subsequent outbreaks involve fresh produce—especially carrots—along with milk and water (Figure 2a) [48]. Foodborne exposure may include elevated bacterial concentrations (10^7^–10^9^ CFU/mL), above the estimated human infectious dosage of 10^4^–10^6^ CFU/mL. Moreover, companion animals like dogs and cats can excrete pathogenic *Y. enterocolitica* in their feces, posing a transmission risk to humans, especially to small children in close proximity to domestic pets [49].

Familial aggregation and environmental factors may influence the emergence of CD and *Y. enterocolitica* infections in individuals. These findings correspond with the research conducted by Van Kruiningen et al. [50], who examined familial CD in Belgium and discovered that environmental factors, including the use of unpasteurized dairy products and undercooked meat, particularly pig, are likely to precipitate CD in genetically susceptible individuals. The findings support the hypothesis that foods with a high risk of *Yersinia* contamination could trigger CD.

Epidemiological studies show that infections caused by *Y. enterocolitica* and *Y. pseudotuberculosis* exhibit a clear seasonal pattern, with incidence rates peaking during the colder months [51]. This aligns with the organism’s ability to thrive at cooler temperatures and its common reservoir in pigs slaughtered during colder seasons. However, whether this seasonal pattern influences the onset or exacerbation of IBD remains unclear, with no consistent evidence linking seasonal *Yersinia* infections to IBD disease activity.

## 4. Interplay Between *Yersinia* and Mucosal Immunity in IBD

Intraepithelial cells (IECs), integral to the gut barrier, maintain intestinal integrity by facilitating the flow of vital ions, minerals, and water while preventing the passage of bacterial toxins and infections [52, 53]. In IBD, compromise of the gastrointestinal lining results in reduced epithelial integrity, triggering a cascade of pathogenic immune responses [54]. The process includes dysregulated immune cell activation, an imbalance between proinflammatory cytokines—such as integrins, interferons (IFNs), and interleukins (IL‐1, IL‐6, IL‐12, IL‐17, IL‐18, IL‐21, and IL‐23), along with tumor necrosis factor alpha (TNF‐α)—and anti‐inflammatory mediators, including IL‐10, IL‐35, and transforming growth factor‐beta (TGF‐β) [54]. Enhanced intestinal permeability facilitates the translocation of bacteria and their metabolites across the epithelial lining, triggering inappropriate immune responses and sustaining mucosal inflammation [55]. For instance, pathogens such as *Y. enterocolitica*, *Campylobacter jejuni* (*C. jejuni*), and AIEC exploit the compromised epithelial barrier, leading to persistent inflammation and mucosal damage [28, 56, 57]. Moreover, altered immune cell response to a dysbiotic microbial association, along with inappropriate or excessive T‐cell activation, intensifies the chronic inflammation and tissue damage typical of CD and UC [54, 58]. It is noted that prolonged activation of innate responses and dysregulation between IECs and unicellular phagocytes, which secrete critical proinflammatory cytokines, may precipitate chronic inflammation in IBD [59]. Furthermore, CD is frequently associated with an elevated immune response, driven primarily by T helper cell type 1 (Th1) and Th17 cells, characterized by increased secretion of cytokines such as IFN‐γ, IL‐12, IL‐17, and IL‐23 [60]. Conversely, UC is generally associated with a Th2/Th9‐mediated response characterized by increased levels of IL‐5, IL‐9, and IL‐13 [61]. Th1‐ and Th17‐mediated responses promote macrophage activation, neutrophil recruitment, and antibacterial immunity, thereby contributing to host defense against enteric pathogens such as *Yersinia* [62–64]. In contrast, Th2‐ and Th9‐mediated responses are primarily associated with mucosal repair, epithelial barrier maintenance, and immune regulation [63–65]. Dysregulation of the balance between these effector pathways is thought to contribute to the distinct immunopathological features of CD and UC [64]. Although these distinctions are beneficial, it is crucial to recognize that all these cytokines are present in the inflamed mucosa, albeit in differing quantities (Table S1).

The genus *Yersinia* has been linked to the onset and progression of IBD, primarily through its interactions with the human immune system and gut microbiome [66]. These interactions may trigger innate immune responses that identify pathogen‐associated molecular patterns (PAMPs) through pattern recognition receptors (PRRs) [67]. These PRRs, such as those within the nucleotide‐binding leucine‐rich repeat (NLR) family proteins, including NLR‐containing protein 3 (NLRP3), NLR family CARD domain‐containing protein 4 (NLRC4), and pyrin, form inflammasomes in response to diverse bacterial toxins and components. Upon stimulation, inflammasomes recruit caspase‐1, which processes proinflammatory cytokines IL‐1β and IL‐18 into their active and functional forms. This event triggers pyroptosis—a destructive, regulated mode of necrotic and inflammatory cell death designed to eliminate pathogens—characterized by caspase activation, cytokine release, and membrane disruption [68]. Furthermore, non‐canonical inflammasomes, like caspase‐4/5/11 inflammasome, can be activated by cytosolic LPS [69]. Persistent inflammasome activation promotes excessive maturation of IL‐1β and IL‐18, thereby amplifying intestinal inflammation through enhanced leukocyte recruitment, epithelial barrier dysfunction, and sustained immune activation. Consequently, dysregulated inflammasome signaling is increasingly recognized as a key driver of chronic mucosal inflammation in IBD [70–72].

Enteropathogenic *Yersinia* species, in contrast to internal infections such as *Salmonella Typhi* and *Brucella*, employ various strategies to evade host immune responses and establish infection [73]. *Yersinia* regulates the expression of its virulence components to avoid immune detection and adapt to environmental fluctuations in temperature [74]. Héchard et al. [75] demonstrated that *Yersinia* modulating protein A (YmoA) serves as a pivotal stress sensor, regulating virulence and metabolism in response to variations in temperature, and osmolarity. At reduced temperatures, they produce flagellin and hexa‐acylated LPS, both of which are powerful toll‐like receptor (TLR) agonists [76]. However, at elevated temperatures within the mammalian host, they downregulate the flagellin expression and generate a less immunogenic variant of LPS. This temperature‐sensitive control enables it to evade early detection by the host immune system [77]. Once the bacteria penetrate the small intestine, they adhere to and infiltrate the specialized microfold (M) cells of the epithelium. *Y. enterocolitica* and *Y. pseudotuberculosis* exhibit various adhesins and virulence factors that may lead to dissemination to the mesenteric lymph nodes or other extraintestinal tissues, such as the spleen, liver, and/or lungs, contingent upon the infected individual’s underlying health (Figure 2b–d). To successfully disseminate and survive in the harsh host environment, such as the acidic stomach, *Yersinia* produces urease [78].

**Figure 3:**
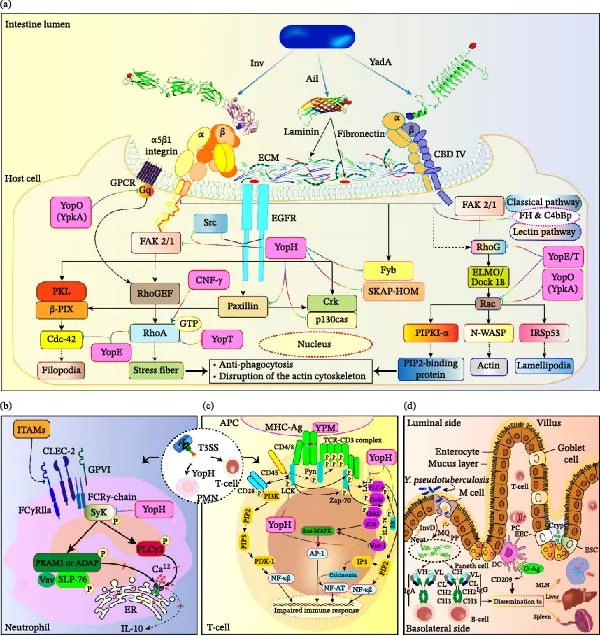
Multifaceted mechanisms of host cell entry, immune evasion, and dissemination mediated by *Yersinia* virulence factors. (a) Colonization begins when *Y. enterocolitica* and *Y. pseudotuberculosis* adhere to and penetrate the M cell epithelium over Peyer’s patches (PP). Attachment is mediated by adhesins (Ail, Inv, and YadA); YadA and Ail also bind C4b‐binding protein, thereby blocking the classical and lectin complement pathways, and ultimately all complement activation routes. After entering PP, bacteria multiply extracellularly, provoking strong inflammation. Yop proteins reshape host cells: YopE and YopT disrupt actin by inactivating or cleaving RhoA; YpkA/YopO inhibits Rho GTPases or phosphorylates Gαq and actin‐binding proteins, altering cytoskeleton, and inducing pyroptosis. YopH dephosphorylates FAK, paxillin, p130‐Cas, SKAP‐HOM, Fyb, and Pyk2, crippling integrin signaling. *Y. pseudotuberculosis* also secretes CNF‐γ, which locks Rho GTPases in an active state, activates STAT3, lowers iNOS, and suppresses the inflammasome, fostering a permissive niche. (b) In human PMNs, YopH dephosphorylates SKAP‐HOM, PRAM‐1, SLP‐76, PLCγ2, and Vav1, blocking integrin‐dependent spreading, degranulation, ROS production, cytokine (IL‐10) release, calcium signaling, and phagocytosis, thereby protecting the bacteria. (c) In T cells, YopH dephosphorylates LAT, preventing recruitment of PLCγ2, Vav‐1, and Lck, and thus halting TCR signaling. In contrast, YPM binds MHC II, hyperactivating innate cells and T cells and inducing a surge of proinflammatory cytokines such as IL‐4. (d) *Y. pseudotuberculosis* engages in two key activities to promote systemic spread and immune modulation: it exploits its LPS core to bind CD209 on dendritic cells and macrophages, facilitating dissemination to MLNs, liver, and spleen; and it expresses the temperature‐regulated adhesin InvD, which preferentially binds IgG and IgA antibodies bearing VH3/VK1 domains, potentially modulating mucosal antibody responses in the gut.

*Yersinia* initiates an infection by invading the gut epithelium through the production of three primary virulence factors: Inv, attachment invasion locus (Ail), and *Yersinia* adhesin A (YadA) (Figure 3a) [79]. Following its invasion of the small intestine, it penetrates lymphoid follicles, particularly M cells, which facilitate bacterial translocation across the mucosal barrier. In the broader context of IBD, epithelial barrier dysfunction is characterized by disruption of tight junction proteins, depletion of the mucus layer, dysregulated antimicrobial peptide production, and Paneth cell dysfunction, all of which contribute to impaired mucosal defense and chronic intestinal inflammation [80–82]. In addition to facilitating epithelial invasion, *Yersinia* further compromises intestinal barrier integrity by disrupting tight junction proteins, including occludin, claudins, tricellulin, and ZO‐1, thereby increasing epithelial permeability [83].

In terms of the binding site, *Y. pseudotuberculosis* demonstrates a superior binding affinity for fibronectin, whereas *Y. enterocolitica* shows a heightened affinity for collagen and laminin [79]. Besides acid tolerance, *Yersinia* utilizes immune evasion mechanisms by producing OMPs such as YadA and Ail, which engage with complement system inhibitors, including C4b‐binding protein and factor H, thus hindering the host’s immunological response [79]. Furthermore, bacteria employ strategies to survive within phagocytic cells by producing virulence factors such as OmpR, global stress requirement (GsrA), and superoxide dismutase (SodA), which are crucial for alleviating oxidative stress and other antimicrobial defenses within the host cell [74].

### 4.1. Secretion Systems and Their Multifaceted Roles in the Pathogenicity of *Yersinia*

The bacteria utilize a common type III secretion system (T3SS) to translocate chromosomal and plasmid‐encoded effector proteins, known as *Yersinia* outer proteins (Yops), into host cells, where they subvert immune signaling and promote bacterial survival [84]. The principal molecular targets and immunomodulatory functions of T3SS effector proteins are summarized in Table S2. Among these effectors, YopE and YopT inactivate Rho GTPases, disrupting cytoskeletal organization, impairing phagocytosis, and attenuating host immune responses (Figure 3a) [85, 86]. YopO (YpkA in *Y. pseudotuberculosis*), a serine/threonine kinase, contributes to *Yersinia* virulence by disrupting Rho GTPase signaling, leading to cytoskeletal rearrangement, impaired phagocytosis, and suppression of host immune responses [87–92]. YopH, a protein tyrosine phosphatase, further subverts host immunity by dephosphorylating key signaling molecules involved in phagocytosis and leukocyte activation, thereby inhibiting calcium signaling, reactive oxygen species (ROS) production, and neutrophil antimicrobial functions (Figure 3a–c) [93, 94]. In addition, YopH limits bacterial uptake by M cells, facilitating immune evasion and bacterial persistence [95].

In *Y. pseudotuberculosis*, additional virulence factors, including cytotoxic necrotizing factor (CNF‐γ), disrupt Rho GTPase activity, thereby affecting cellular signaling pathways such as phosphoinositide 3‐kinase (PI3K)/protein kinase B (Akt)/NF‐κβ and signal transducer and activator of transcription 3 (STAT3) (Figure 3a) [96, 97]. Furthermore, *Y. pseudotuberculosis*‐derived mitogen (YPM) directly binds to class II major histocompatibility complex (MHC II) molecules, effectively activating T cells, including hepatotoxic CD4^+^ T cells (Figure 3c) [98]. Also, *Y. pseudotuberculosis* synthesizes InvD, a virulence component that selectively binds to host IgG/IgA antibodies with VH3/VK1 domains, likely targeting specific B‐cell subsets and modifying antibody function and disease development (Figure 3d) [99].

**Figure 4:**
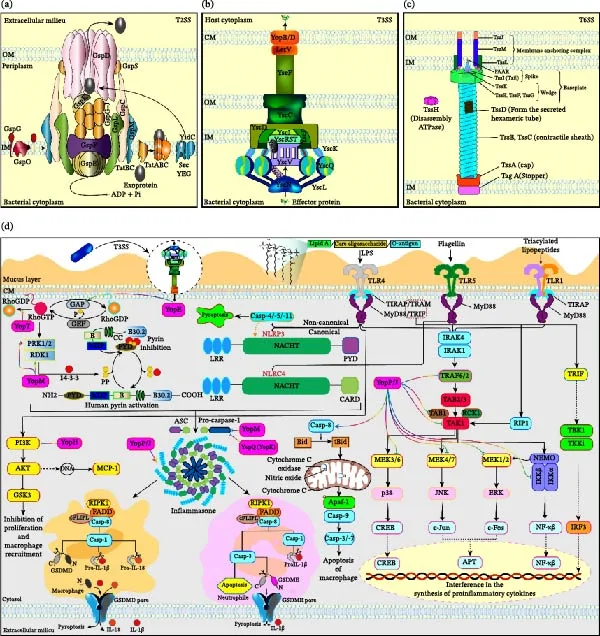
Schematic representation of *Yersinia* secretion systems and their roles in modulating host signaling pathways. (a) In *Yersinia*, the T2SS secretes exoproteins synthesized in the cytoplasm, moved to the periplasm via the SecYEG translocase, and exported through a GspD channel. GspO removes the prepilin leader from GspG, which assembles with GspF‐L‐E; GspE supplies ATP. Substrate choice likely involves GspC–GspD. GspS stabilizes the channel in some species. (b) At 37°C in rich medium, the Ysc T3SS builds its basal body (YscC/D/J), polymerizes a YscF needle, inserts the YopB/D–LcrV translocon, and injects Yop effectors into host cytoplasm. (c) The T6SS, phage‐like and built from 13 core subunits, comprises a membrane complex, baseplate, and injection apparatus. The injector contains the sheath (TssBC), tail tube (Hcp/TssD), and spike (VgrG/TssI plus PAAR proteins), whose release marks T6SS activity. (d) *Yersinia* effectors dampen inflammation. YopP/J blocks IKKβ and MKKs, preventing NF‐κB and MAPK activation, halting transcription of pro‐survival and proinflammatory genes and inducing macrophage apoptosis. YopT and YopE inactivate RhoA, lowering PKN1/2 and triggering pyrin‐inflammasome assembly. LPS activates canonical and non‐canonical inflammasome pathways; caspase‐1 cleaves GSDME/GSDMD, releasing IL‐1β and IL‐18 and causing pyroptosis. YopM phosphorylates pyrin via PKN1/2–RSK1, binds and sequesters caspase‐1, depletes NK cells, and alters IL‐18. YopQ/K restricts effector translocation, limiting PAMP entry and caspase‐1 activation. YopH dephosphorylates Akt, inactivating PI3K/Akt signaling and reducing MCP‐1–mediated macrophage recruitment.

In addition to the T3SS, the type II secretion system (T2SS) is a crucial component for the maintenance and pathogenicity of *Yersinia* (Figure 4a,b) [100]. In *Y. enterocolitica*, the Yts1 T2SS is essential for virulence, facilitating dispersion throughout the host and survival in critical organs such as the mesenteric lymph nodes, liver, and spleen [101]. Furthermore, the T6SS has been recognized as a significant pathogenicity factor in numerous *Yersinia* species (Figure 4c) [102]. This intricate apparatus is an essential mechanism in bacterial pathogenesis, enhancing cytotoxicity, facilitating intracellular persistence, and contributing to effective host spread [103].

### 4.2. Dysregulation of Host Immune Responses by Virulence Factors of *Yersinia*

Apart from the aforementioned factors, *Yersinia* spp. employs other virulence factors to subvert host immunity, the principal molecular targets and immunomodulatory functions of which are summarized in Table S2. YopE and YopT significantly modulate inflammation via regulating the release of caspase‐1 and IL‐1β, in addition to inflammasome activation (Figure 4d) [104]. YopE plays a complex function in *Yersinia* infections by provoking an immune response while concurrently obstructing bacterial absorption by specific M cells [95]. YopP (YopJ in *Y. pseudotuberculosis*) is an acetyltransferase effector that suppresses host immune responses by targeting MAPK and NF‐κB signaling pathways, thereby reducing proinflammatory cytokine production and promoting macrophage apoptosis [93, 105]. In addition to its anti‐inflammatory activity, YopP/J modulates cell death pathways by influencing apoptosis and pyroptosis through caspase‐dependent mechanisms, including RIPK1–caspase‐8‐mediated GSDME activation and IL‐1β release [106–110]. Additionally, YopJ has been demonstrated to inhibit prostaglandin E2 (PGE2) production, hence further influencing host immunity [111]. Despite these findings, the molecular factors that regulate the transition between apoptotic and pyroptotic pathways in response to YopP/J activity remain a subject of current research (Figure 4d). YopM contributes to immune evasion by disrupting inflammasome activation through interactions with host kinases, thereby limiting inflammatory responses and promoting bacterial persistence [112]. YopQ (YopK in *Y. pseudotuberculosis*), a unique virulence factor shared throughout pathogenic *Yersinia* species, is essential for inhibiting inflammasome activation and evading innate immune responses [113], specifically neutrophil‐mediated phagocytosis, by targeting antiphagocytic effector proteins (Figure 4d) [114].

In *Y. pseudotuberculosis*, TsTYp exerts diverse effects on immune cells via cyclic adenosine monophosphate (cAMP)‐dependent activation of protein kinase A and downstream targets such as extracellular signal‐regulated kinase 1/2 (ERK1/2) and phosphatidylinositol 3‐kinase (PI3K)/protein kinase B (AKT) [113, 115]. It modulates phagocytic activity in both neutrophils and monocytes, impacting ROS production and apoptosis. TsTYp regulates cAMP concentrations in a dose‐dependent manner, inhibiting ROS formation in monocytes at low doses—potentially facilitating immune evasion—while augmenting ROS creation in neutrophils [116]. Furthermore, TcpYI possesses a C‐terminal hydrophobic domain characteristic of the transport inhibitor response 1 (TIR1) domain family, which promotes intracellular survival of *Y. pseudotuberculosis* within macrophages and in mouse spleens [117]. Previous studies have shown that toxin‐coregulated pili (TCP)‐containing bacteria often suppress host defenses by targeting TLR4 signaling, a key pathway in pyroptosis induction [118, 119]. Particularly, TcpYI directly binds to TIR, suggesting a mechanism to block TLR4 signaling and potentially protect host cells from nicotinamide adenine dinucleotide (NAD^+^) depletion via its NAD^+^‐hydrolase activity. Moreover, *Y. pseudotuberculosis* can also exploit host cell receptors, such as CD209, to facilitate dissemination to systemic organs. This interaction promotes bacterial spread to lymph nodes, the kidney, the spleen, and the liver [120], resulting in consequences such as septic shock, acute organ damage, abscess development, and splenic infarction, especially in individuals with liver disease or compromised immunity [121, 122].

The aforementioned genetic factors, encompassing specific mutations and the dysregulation of cytokines such as IL‐1, TNF‐α, IL‐6, monocyte chemoattractant protein‐1 (MCP‐1), IL‐10, IFN‐γ, and TGF‐β1, alongside the absence of TLR1 and TLR5 and the presence of the immunomodulatory protein galectin‐1 [123–125], which hinders bacterial clearance, collectively foster chronic inflammation in the abdominal cavity of individuals with IBD. While bacterial invasion is deemed significant in the pathophysiology of IBD (Table 1), the molecular specifics regarding the subsequent weakening of the intestinal barrier remain mostly unknown.

**Table 1:** Properties of the chromosomal and plasmid‐encoded virulence factors in *Yersinia* species.

Genetic location	Virulence determinant	Size (kDa)	Type of protein	Target	Function	Refs.
Chromosomal	InvA in *Yersinia* spp.	92	• Integral OMP	• α3β1, α4β1, α5β1, α6β1, αvβ1 integrins	• Facilitates cell adhesion • Promotes bacterial translocation into M cells • Enables colonization of Peyer’s patches • Triggers proinflammatory responses	[79]
	InvB‐E in *Y. pseudotuberculosis*	NA	• OMP	• β1 integrins • IgG/IgA VH3/VK1 (InvD)	• InvB: facilitates colonization • InvC: enhances adherence • InvD/E: roles remain unidentified	[79, 99]
	Ail	17	• OMP	• C4b2a, factor H	• Facilitates attachment • Promotes invasion • Collaborates with YadA for serum resistance	[77]
	YstA‐C	3.5–6	• Heat‐stable enterotoxin A	• Host cell actin	• Activates guanylate cyclase • Elevates cyclic GMP levels in intestinal cells	[126]
	YspA‐F, YspI, YspK‐N, YspP, YspY	Variable	• T3SS	• Cytoplasmic membrane	• Regulates Yops secretion • Modulates immune response • Facilitates epithelial invasion	[79]
	Yersiniabactin	482	• Siderophore (catechol‐type)	• Psn/FyuA	• Facilitates iron acquisition in the host • Highly expressed in the peritoneum • Moderately expressed in the spleen	[127]
	Urease	61.09	NA	• Urea	• Confers acid resistance in stomach	[79, 128]
	Yts1 and Yts2	NA	• T2SS	NA	• Essential for systemic dissemination • Not involved in initial infection of Peyer’s patches	[129]
	T6SS	NA	• Secretion system	NA	• Delivers toxic effectors to host cells and competing bacteria • Facilitates immune evasion • Contributes to biofilm formation • Involved in autophagy modulation	[103]
	LPS	48.85	• Endotoxin	• TLR4	• Triggers cytokine production via TLR4 • Acts as a potent immune activator	[130]
	CNF_*Y*_ in *Y. pseudotuberculosis*	114	• Cytotoxin	• RhoA • Rac1	• Induces multinucleation • Promotes DNA replication • Inhibits inflammasome activation • Stimulates IL‐6 and IL‐8 production	[131–134]
	YPM in *Y. pseudotuberculosis*	14	• Super‐antigen	• T cells • Macrophages	• Potently activates T cells	
	TsTYp in *Y. pseudotuberculosis*	45	• Toxin family	• Neutrophils • Monocytes	• Modulates ROS and cAMP levels in phagocytes • Disrupts vascular integrity	
	TcpYI in *Y. pseudotuberculosis*	24.6	• Effector protein	• TLR4	• Induces pathogenicity independently of the pYV plasmid • Inhibits TIR signaling	
Virulence plasmid (pVY)	YadA	160.240	• Trimeric autotransporter	• Collagen I, II, IV • Laminin • Vitronectin	• Mediates adhesion • Induces cytokine production • Confers resistance to complement • Provides serum resistance	[79]
	LcrV (V‐antigen), PopB and PopD, other Ysc proteins	Variable	• T3SS	• Cytoplasmic membrane	• Injects Yop proteins into host cells • Disrupts phagocytosis and apoptosis • Suppresses host immune responses	[79, 135]
	YopE	22.95	• T3SS	• RhoA • Rac1 • Cdc42	• Inhibits Rho GTPases • Modulates effector translocation • Influences inflammasome activation	[79, 136]
	YopT	36.37		• RhoA • Rac1 • Cdc42	• Disrupts actin cytoskeleton • Induces pyroptosis • Interacts with YopE	
	YopO in *Y. enterocolitica* YpkA in *Y. pseudotuberculosis*	81.7		• RhoA • Rac1 and Rac2	• Disrupts phagocytosis • Alters protein phosphorylation • Suppresses immune responses	
	YopH	51		• FAK • P130Cas • Lck • Fyb • SKAPHOM • p85 • Paxillin	• Inhibits neutrophil phagocytosis • Inhibits neutrophil degranulation • Promotes colonization	
	YopP in *Y. enterocolitica* YopJ in *Y. pseudotuberculosis*	31–32		• MAKKs • TRAF6/2 • IKKβ	• Blocks MAPK, TAK1, and NF‐κB signaling pathways • Induces cell death • Inhibits PGE2 production	
	YopM	42–54		• PRK2 • RSK1 • Caspase‐1	• Modulates the immune response • Induces IL‐10 production • Triggers apoptosis in immune cells	
	YopQ in *Y. enterocolitica* YopK in *Y. pseudotuberculosis*	20.83		Inflammasome	• Controls Yop protein translocation • Inhibits inflammasome activation	

Abbreviations: cAMP, cyclic adenosine monophosphate; CNFYᵀY, cytotoxic necrotizing factor Y; FAK, focal adhesion kinase; Fyb, Fyn‐binding protein; IgG/IgA, immunoglobulin G/A; IKKβ, IκB kinase beta; IL, interleukin; Lck, lymphocyte‐specific protein tyrosine kinase; LcrV, low calcium response V antigen; LPS, lipopolysaccharide; MAKKs, MAPK kinase kinases; MAPK, mitogen‐activated protein kinase; NF‐κB, nuclear factor kappa‐light‐chain‐enhancer of activated B cells; OMP, outer membrane protein; P130Cas, Crk‐associated substrate; p85, regulatory subunit of PI3K; PGE2, prostaglandin E2; PopB, PopD, pore‐forming proteins B and D (components of T3SS); PRK2, protein kinase C‐related kinase 2; pYV, plasmid *Yersinia* virulence; RhoA, Rac1, Rac2 and Cdc42, members of the Rho family of GTPases; ROS, reactive oxygen species; RSK1, ribosomal S6 Kinase 1; SKAPHOM, Src kinase‐associated phosphoproteins SKAP55 homolog; T2SS, type II secretion system; T3SS, type III secretion system; T6SS, type VI secretion system; TIR, toll/interleukin‐1 receptor; TLR4, toll‐like receptor 4; TRAF6/2, TNF receptor‐associated factor 6/2; VH3/VK1, variable heavy chain 3/variable kappa chain 1; YadA, *Yersinia* adhesin A; Yop, *Yersinia* outer protein; Ysc, *Yersinia* secretion (protein family).

## 5. Classification Methods and Genetic Attributes in IBD

In 2003, a dedicated group of researchers established a classification system for IBD that integrates clinical, genetic, and serological information. The Vienna and Montreal classification systems describe CD based on age of onset, geographic distribution, and disease behavior. The Montreal classification system for CD has introduced a new category for early‐onset disease, defined as diagnosis at 16 years of age or younger. Serological markers, including anti‐neutrophil cytoplasmic autoantibody (p‐ANCA) and anti‐*Saccharomyces cerevisiae* antibody (ASCA), have been thoroughly investigated in IBD. Although they may be beneficial in some situations, their restricted sensitivity and inconsistent prevalence among patients with IBD limit their diagnostic effectiveness.

**Figure 5:**
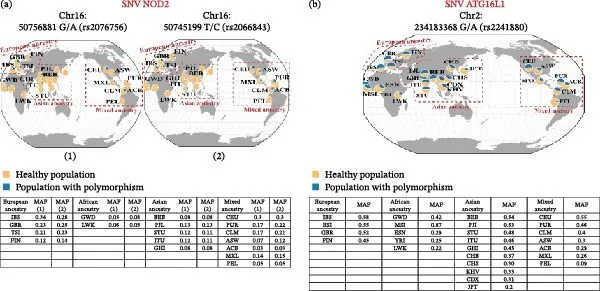
The frequency of NOD2 (a) and ATG16L1 (b) between different populations in all over the world. ACB, African Caribbean in Barbados; ASW, African ancestry in southwest United States; BEB, Bengali in Bangladesh; CDX, Chinese Dai in Xishuangbanna, China; CEU, CEPH/Utah residents (CEPH) with northern and western European ancestry; CHB, Han Chinese in Beijing, China; CHS, Han Chinese South; CLM, Colombian in Medellin, Colombia; ESN, Esan in Nigeria; FIN, Finnish in Finland; GBR, British in England and Scotland; GIH, Gujarati Indians in Houston, Texas; GWD, Gambian in Western Division, The Gambia – Mandinka; IBS, Iberian populations in Spain; ITU, Indian Telugu in the United Kingdom; JPT, Japanese in Tokyo, Japan; KHV, Kinh in Ho Chi Minh City, Vietnam; LWK, Luhya in Webuye, Kenya; Maf, minor allele frequency; MSL, Mende in Sierra Leone; MXL, Mexican ancestry in Los Angeles, California; NRC, not rate calculated; PEL, Peruvian in Lima, Peru; PJL, Punjabi in Pakistan; PUR, Puerto Rican in Puerto Rico; SNV, single nucleotide variant; STU, Sri Lankan Tamil in the United Kingdom; TSI, Toscani in Italy; YRI, Yoruba in Ibadan, Nigeria. The figures were illustrated utilizing “Geography of genetic variants browser”.

As of now, more than 150 genetic loci have been associated with susceptibility to CD. Genome‐wide association studies (GWAS) across many populations have consistently identified single‐nucleotide polymorphisms (SNPs) in genes such as caspase recruitment domain‐containing protein 15/nucleotide‐binding oligomerization domain‐containing protein 2 (*Card15/Nod2*), immunity‐related GTPase M (*Irgm*), interleukin 23 receptor (*Il23r*), autophagy‐related 16‐like 1 (*Atg16l1*), and leucine‐rich repeat kinase 2 (*Lrrk2*) as significant risk factors for the condition [137, 138]. Genetic polymorphisms in the NOD2 gene (rs2066843 and rs2076756), located on chromosome 16, impair the recognition of the bacterial component muramyl dipeptide (MDP) (Figure 5a) [139]. NOD2 is crucial for maintaining intestinal health by regulating immune responses to beneficial gut microorganisms. Beyond its role in sensing invading pathogens, NOD2‐mediated recognition of MDP is critical for maintaining immune tolerance to the commensal gut microbiota. Under homeostatic conditions, NOD2 fine‐tunes innate immune signaling, restrains excessive NF‐κB activation, promotes Paneth cell antimicrobial peptide secretion, and preserves host–microbiota mutualism. Consequently, loss of NOD2 function disrupts these regulatory mechanisms, leading to impaired tolerance to commensal bacteria, microbial dysbiosis, and aberrant mucosal immune activation, thereby fostering the chronic intestinal inflammation that characterizes CD [24, 140, 141]. NOD2 is also implicated in autophagy, a cellular mechanism that eliminates intracellular pathogens. Inadequacies in NOD2‐mediated autophagy may lead to bacterial buildup in the gut epithelium, hence intensifying inflammation [142].

Moreover, extensive research has been conducted on the T300A polymorphism in the ATG16L1 autophagy gene (A > G SNP; chr2; rs2241880), which is associated with CD (Figure 5b) [23, 143]. Cadwell et al. (2008) demonstrated that Paneth cells lacking ATG16L1 exhibit deficiencies in granule exocytosis and gene expression, particularly concerning genes that are upregulated in the contexts of lipid metabolism and inflammation. Individuals possessing the ATG16L1 risk allele exhibit cellular abnormalities similar to those observed in patients with CD [144]. GWAS have repeatedly identified ATG16L1 T300A as a principal susceptibility gene for CD, particularly in Caucasian populations [145]. Researchers also recognize the G allele of this SNP as a low‐penetrance risk factor, suggesting a complex interaction between genetic and environmental influences in the onset of CD [146]. The frequency rate of ATG16L1 and NOD2 among various populations aligns with the global distribution of yersiniosis, as illustrated in Figures 2 and 5. This genetic diversity in North America, Asian nations, including China and India, and northern Europe highlights the intricate interaction between genetic and environmental influences in the onset of IBD.

## 6. Relationship Between *Yersinia* Infection and IBD

Individuals with IBD commonly develop enteric infections that typically induce gut microbiota dysbiosis. Although the precise etiology of IBD remains uncertain, genetic and environmental factors play key roles in the disease pathophysiology and progression [5]. An impaired intestinal environment characterized by inflammation facilitates the proliferation of detrimental microbes and functionally modified commensal bacteria, referred to as pathobionts, which may exhibit pathogenic activity under adverse conditions. In addition to microbial variation, IBD demonstrates differences in illness distribution. In East Asia, 50%–70% of individuals exhibit ileocolonic engagement, while studies in Western cultures indicate similar participation across the ileal, colonic, and ileocolonic regions.

The etiology of UC is multifaceted, with increasing evidence emphasizing the significance of microbial dysbiosis—specifically, *Y. enterocolitica* infection—due to its immunomodulatory effects and tissue‐invasive capabilities [147]. Moreover, infection with *Y. enterocolitica* O:3 has been identified as a potential initiating component in the pathogenesis of UC, highlighting the importance of microbial triggers in the disease’s origin and progression [66]. Several case reports have detected *Yersinia* spp. in the intestinal tissues of individuals with CD, indicating a potential association (Table 2).

**Table 2:** A summary of case reports on *Yersinia* infection mimicking IBD: clinical findings, diagnostic methods, and treatment.

Author (year)	Country	Age	Gender	Tests	Specimen	Diagnostic method	Species/serotype	Treatment	Final diagnosis (CD/UC/*Yersinia*)	Refs.
Revés et al. (2024)	Portugal	48 years	F	• Hb: increased from 9.8 to 13.5 g/dL • CRP: decreased from 45.27 to 0.19 mg/dL • FCP: decreased from 2000 to 48 μg/g • PLT: decreased from 465 to 316 × 10^9^/L • *Y. enterocolitica* O:3 Ab: positive • Stool and blood cultures: negative	• Serum • Stool • Blood	• Serology (ELISA) • Stool and blood culture • CT scan • Colonoscopy with biopsy • MR‐enterography	*Y. enterocolitica*/O:3	• Ceftriaxone • Metronidazole: Initially IV, then switched to oral for 7 days	–/–/+	[148]
Doshi et al. (2024)	United States	77 years	F	• Hb: 12.6 g/dL (normal) • WBC count: 8.8 × 10^9^/L (normal) • PLT: 211 × 10^9^/L (normal) • ESR: 15 mm/h (normal) • CRP: 3.7 mg/L (mildly elevated) • K^+^: 2.8 mmol/L (low–hypokalemia) • Stool markers: not performed	• Blood • Stool	• PCR GI panel • Clinical history (colonoscopy performed in 2021) • Symptoms	*Y. enterocolitica*/NS	• IV Solu‐Medrol: discontinued • IV fluids and supportive care: provided • Humira and oral prednisone: resumed	–/+/+	[149]
Filipiuk et al. (2024)	Poland	47 years	M	• Stool calprotectin: 410 mg/kg (elevated) • CRP: 1.1 mg/dL (elevated) • Routine laboratory tests: largely normal	• Stool • Blood	• Stool and blood cultures • Serology panel • Colonoscopies with biopsies • Tendon ultrasonography • Spine/pelvis imaging	ND	• Multiple lines of treatment • Current therapy: dual biologic therapy: ustekinumab and adalimumab, methotrexate (in combination) • Previously used treatments: steroids, mesalazine, infliximab, and tofacitinib	–/+/–	[150]
Braga et al. (2024)	Brazil	23 years	M	• PCR: positive for *Yersinia* spp. • Lymph node biopsy: granulomatous lymphadenitis with central necrosis • AFB test: negative	• Lymph node biopsy	• CT scan • Colonoscopy with biopsy • Histopathology • PCR	*Yersinia* spp./NS	• Initial antibiotics: ciprofloxacin and metronidazole • Escalated to: piperacillin with tazobactam • Then switched to: meropenem, administered for 10 days	–/–/+	[151]
Marubashi et al. (2023)	Japan	~28 years	F	• CRP: 0.7 mg/dL (mildly elevated) • *H. pylori* antibody: positive	• Ileal biopsy	• Colonoscopy with biopsy and culture • Video capsule endoscopy	*Y. enterocolitica*/NS	• Levofloxacin: 500 mg once daily for 7 days	–/–/+	[152]
Sood et al. (2023)	United States	45 years	M	• CRP: 11 mg/L (elevated) • GI pathogen panel: positive for *Yersinia*	• Stool	• CT enterography • Colonoscopy • Biopsy • GI pathogen panel	*Yersinia*/NS	• Ciprofloxacin: administered for 2 weeks	–/–/+	[153]
Ali et al. (2023)	Saudi Arabia	27 years	F	Initial findings:• Hb: 9.6 g/dL • ESR: 90 mm/h • CRP: 110 mg/L • Stool occult blood: positive • Stool cultures: negative ED visit: • WBC: 38 × 10^9^/L • CRP: 341 mg/L • ESR: 43 mm/h	• Stool • Blood	• Stool culture only performed (no PCR or serology) • Note: possible diagnostic bias due to limited testing	ND	• Medications used prior to surgery: mesalamine, prednisolone, azathioprine, and adalimumab • Surgical intervention: total colectomy with end ileostomy	+/–/–	[154]
Norrito et al. (2021)	Italy	79 years	F	• WBC count: increased from 15.9 to 20.7 × 10^3^/µL • CRP: increased from 40 to 97 mg/L • FCP: >300 mg/kg (elevated) • Blood cultures: positive for *Y. enterocolitica*	• Blood	• CT and MRI of the abdomen • PET‐CT scan • Colonoscopy with biopsy • Stool tests • Blood cultures	*Y. enterocolitica*/NS	• Empiric therapy: ciprofloxacin and metronidazole • Switched to targeted therapy: cefixime and doxycycline (duration: 1 month)	–/–/+	[155]
Cian et al. (2020)	Argentina	12 years	M	• WBC count: 14,620/mm^3^ (elevated) • Hb: 12.3 g/dL (within normal range) • ESR: 35 mm/h (elevated) • CRP: 2.5 mg/dL (elevated) • FCP: normal	• Biopsy culture • Intestinal fluid analysis	• Colonoscopy • GI panel (filmArray) • Biopsy culture • CT scan • Histology	*Y. enterocolitica*/NS	• Symptomatic management only • No antibiotics reported	–/–/+	[156]
Joung et al. (2019)	South Korea	6 years	M	• WBC count: 16,280/µL (elevated) • ESR: 38 mm/h (elevated) • CRP: 2.26 mg/dL (elevated) • Hb: 10.4 g/dL (low) • Ferritin: 14 ng/mL (low) • Stool FCP: 3000 mg/kg (markedly elevated)	• Stool • Biopsy culture	• Abdominal US • CT scan • Colonoscopy with biopsy • Stool multiplex RT‐PCR	*Y. enterocolitica*/NS	• Initial treatment: EEN and mesalazine (discontinued) • Current approach: supportive care	−/−/**+**	[157]
Franczak et al. (2018)	Poland	38 years	M	• WBC count: 22,000/µL (elevated) • CRP: 164 mg/L (elevated) • Anti‐*Yersinia* ELISA titer: rising (positive) • Stool culture: negative	• Serum • Intestinal tissue • Lymph node biopsy • Stool	• Abdominal US • CT scan • Two exploratory laparoscopies • Histopathology of resected bowel and lymph nodes • ELISA serology • Stool culture	*Yersinia* spp./NS	• Initial medical treatment: mesalazine, steroids, antibiotics • Definitive surgical intervention: right‐sided hemicolectomy, resection of 80 cm of distal ileum	–/–/+	[158]
Almeida et al. (2021)	Brazil	26 years	M	• CT scan: obstructive mass detected • Histopathology: granulomas present, some with suppuration • PCR and IHC for *Yersinia* spp.: negative	• Surgical specimen (intestinal tissue and lymph node biopsy) • Paraffin blocks	• CT scan • Colonoscopy • Histopathology • PCR and IHC	ND	• Surgical intervention: right hemicolectomy • Postoperative course: no maintenance medications used afterward	*+/–/–*	[159]
Wilczyńska et al. (2018)	Poland	20 months	M	• Hb: decreased from 10.4 to 9.2 g/dL • WBC count: increased from 14.1 to 17.6 × 10^6^/µL • CRP: decreased from 3.85 to 0.63 mg/dL • ESR: decreased from 32 to 20 mm/h	• Stool • Colonic biopsy	• Stool and blood cultures • Allergy panel • Abdominal US • Ileocolonoscopy • Histopathology	ND	• Initial treatment (IV): methylprednisolone, ceftriaxone, and metronidazole • Subsequent treatment (oral and supportive): mesalazine, tapering course of steroids, elimination diet, and iron supplementation	*–/+/–*	[160]
Naddei et al. (2017)	Italy	11 years	M	• FCP: 185 µg/g (elevated) • Stool culture: positive for *Y. enterocolitica* O:8 • CBC and CRP: within normal limits	• Stool	• Abdominal US • Ileocolonoscopy with biopsy • Histology • Stool culture • MR enterography	*Y. enterocolitica*/O:8	• EEN: discontinued • TMP‐SMX: administered for 10 days	–/–/**+**	[161]
Warner et al. (2016)	United Kingdom	42 years	M	• WBC count: 11.9 × 10^9^/L (elevated) • Neutrophils: 9.3 × 10^9^/L (elevated) • CRP: 36 mg/L (elevated) • Stool culture: positive for *Y. enterocolitica* • Serology: negative	• Stool • Ileal biopsy • Blood	• CT scan • Ileocolonoscopy • Histopathology • Stool culture • Serology	*Y. enterocolitica*/NS	• Ciprofloxacin: administered for 2 weeks • Initial treatment: modulen and metronidazole (both discontinued)	–/–/**+**	[162]
Azghari et al. (2016)	Morocco	41 years	F	• Hb: 8 g/dL (anemia) • WBC count: normal • Hct: 29% • MCV: 81 fL (normocytic)	• Surgical specimen	• Histopathology: from surgical specimen • CT scan: suggestive of pseudotumor • Stool tests and culture: not mentioned	*Y. enterocolitica*/NS	• Surgical procedure: ileocecal resection • Antibiotic therapy: fluoroquinolones 500 mg orally, twice daily (po bid)	–/–/+	[163]
Botianu et al. (2015)	Romania	64 years	M	• ESR: 32 mm/h (elevated) • Stool cultures (×2): negative • *Yersinia* serology: IgA: 3.2 IU (positive), IgG: 2.3 IU (positive)	• Serum • Stool	• Colonoscopy with biopsies • Histopathology • Abdominal US • Repeat stool cultures • *Yersinia* serology	*Y. enterocolitica*/NS	• Initial antibiotic: rifaximin • Switched to: ciprofloxacin 400 mg orally, twice daily (po bid) for 10 days	–/–/**+**	[164]
Ijichi et al. (2012)	Japan	5 years	F	• CRP: 20.1 mg/dL (elevated) • Paired serum antibodies: positive for *Y. pseudotuberculosis*	• Serum	• Serology • Colonoscopy • Histology • Abdominal US • CT scan • FDG‐PET scan	*Y. pseudotuberculosis*	• Antibiotics: administered (type unspecified) • Clinical outcome: improvement observed by day 9	–/–/+	[165]
Safa et al. (2008)	France	33 years	M	• WBC count: 19.9 × 10^9^/L (elevated) • Neutrophils: 16.0 × 10^9^/L (elevated) • CRP: 29 mg/dL (elevated) • Stool culture: positive for *Y. enterocolitica* biotype 1 A	• Stool • Skin biopsy • Colonic biopsy	• Stool culture • Colonoscopy with biopsy • Histology • Skin biopsy • CARD15/NOD2 gene testing	*Y. enterocolitica/*biotype 1A	• Initial treatment: ceftriaxone • Switched to: prednisone 1 mg/kg/day (due to CD diagnosis) • Clinical outcome: symptoms resolved	+/–/+	[166]
Homewood et al. (2003)	United Kingdom	20 years	F	• Hb: 7.6 g/dL (low–anemia) • Albumin: 19 g/L (low–hypoalbuminemia) • WBC count: 9.7 × 10^9^/L (within normal range) • Serology: positive for *Y. pseudotuberculosis* (titer 1:320) • Stool culture: negative	• Serum • Stool • Surgical specimen	• CT scan • Laparotomy • Histology • Serology • Colonoscopy • Small bowel follow‐through	*Y. pseudotuberculosis*/serotype IV	• Initial surgery: ileal resection with ileostomy • Subsequent procedure: right hemicolectomy • Ongoing care: supportive management	+/–/+	[132]
Tuohy et al. (1999)	United States	3.5 years	F	• ESR: elevated, up to 103 mm/h • CRP: 13 mg/dL (elevated) • Hct: 20% (low) • WBC count: 25,000/µL (leukocytosis) • Anemia: present • Stool culture: positive for *Yersinia*	• Stool	• Abdominal US • CT scan • Colonoscopy • Biopsy • Enteroclysis • Stool culture using CIN agar • Serology	ND	• Current treatment: TMP‐SMX for 14 days • Prior treatments: piperacillin‐tazobactam, metronidazole, and intravenous steroids (tapered)	–/–/–	[167]
Macfarlane et al. (1986)	United Kingdom	10 years	M	• Iron‐deficiency anemia: present • ESR: elevated • *Y. enterocolitica* O:3 antibody titer: • Initially 1:160 • Later decreased to 1:40	• Serum	• *Yersinia* serology: tube‐agglutination test • Small‐bowel barium study • Histology of resected ileum	*Y. enterocolitica*/O:3	• Oral medications: prednisolone, sulfasalazine	+/–/+	[168]
Lachman et al. (1977)	United States	2 years	M	• WBC count: increased to 14,000/µL with left shift • Peripheral smear: toxic granulations present • Stool culture: positive on day 12 • Isolated organism: *Y. enterocolitica*, serotypes 7 and 8	• Stool	• Stool culture • Serology • Sigmoidoscopy • Rectosigmoid biopsy • Barium enema • Electron microscopy	*Y. enterocolitica*/serotype 7/8	IV ampicillin: 200 mg/kg/day	–/–/+	[169]

Abbreviations: (–), negative result; (+), positive result; Ab, antibody; Abdominal US, abdominal ultrasound; AFB, acid‐fast bacilli; Albumin, serum albumin; CBC, complete blood count; CDC, Centers for Disease Control and Prevention; CIN agar, cefsulodin‐irgasan‐novobiocin agar; CRP, C‐reactive protein test; CT scan, computed tomography scan; ED visit, emergency department visit; EEN, exclusive enteral nutrition; ELISA, enzyme‐linked immunosorbent assay; ESR, erythrocyte sedimentation rate; F, female; FCP, fecal calprotectin; FDG‐PET, fluorodeoxyglucose positron emission tomography; Ferritin, iron storage protein; GI, gastrointestinal; Hb, hemoglobin; Hct, hematocrit; IgA, IgG, IgM; immunoglobulin A, G, M; IHC, immunohistochemistry; IU, international units; IV, intravenous; K^+^, potassium; M, male; MCHC, mean corpuscular hemoglobin concentration; MCV, mean corpuscular volume; mg po bid, milligrams by mouth‐twice daily; MR, enterography, magnetic resonance enterography; Na+, sodium; ND, not detected; NS, not specified; PCR, polymerase chain reaction; PET, positron emission tomography; PLT count, platelet count; RBC, red blood cell count; TMP‐SMX, trimethoprim‐sulfamethoxazole; WBC, white blood cell count.

Furthermore, Kallinowski et al. [170] demonstrated the presence of *Yersinia* species in 63% of patients with CD and 46% of patients with UC using culture and polymerase chain reaction (PCR), compared with the control group. Lamps et al. [21] detected pathogenic *Y. enterocolitica* DNA in 31% of bowel resection specimens from patients with CD, whereas all controls were PCR negative. Saebo et al. [66] reported higher serological reactivity to enteropathogenic *Yersinia* among patients with CD than among controls. Knösel et al. [171] identified *Yersinia* species in archived intestinal tissues, whereas Khan et al. [2] detected *Y. enterocolitica* by RT‐PCR in 10.1% of patients with CD, demonstrating a significant association with the disease. Similarly, Le Baut et al. [22] detected *Yersinia* species, including *Y. enterocolitica*, *Y. pseudotuberculosis*, and *Y. intermedia*, in the ileum of ~10% of patients with CD.

Collectively, these findings should be interpreted with caution. Most studies relied primarily on PCR‐based detection of bacterial DNA or serological assays, whereas culture‐based confirmation of viable organisms was rarely achieved, conventional cultures were frequently negative, and sequencing of PCR amplicons was often not performed to confirm target specificity. Consequently, the available evidence largely reflects the detection of bacterial DNA or previous exposure rather than active or persistent infection. In addition, considerable methodological heterogeneity—including differences in specimen type, PCR primer design and target genes, molecular assays, tissue preservation, and study populations—likely contributed to the marked variability in detection rates. Therefore, although these studies suggest a possible association between *Yersinia* and CD, they do not establish a causal relationship or confirm that viable organisms persist within intestinal tissues [21, 30]. Future well‐designed studies integrating standardized molecular techniques with sequencing confirmation and culture‐based detection of viable organisms are required to clarify the pathogenetic role of *Yersinia* in IBD.

Another study demonstrated that *Y. pseudotuberculosis* attaches to β1‐integrin receptors on intestinal epithelial cells through invasin (*inv*), resulting in the formation of large vesicles that encapsulate both the bacteria and the surrounding fluid, including nanoparticles, and facilitate their transport across the epithelial cells via transcytosis. The compromise of the intestinal barrier facilitates the translocation of bacteria and other deleterious substances into the circulation, hence leading to the onset of CD [172]. These observations are consistent with the current ecological model of IBD, in which disease arises from disrupted host–microbiota interactions rather than a single infectious agent [141]. Within this framework, *Yersinia* may function as a pathobiont whose persistence is favored by microbial dysbiosis, epithelial barrier dysfunction, and impaired immune regulation, thereby contributing to chronic mucosal inflammation in genetically susceptible individuals [141, 173].

## 7. Therapeutic Strategies and Antimicrobial Resistance

*Yersinia* gastroenteritis has numerous clinical and pathological similarities with CD, especially in the terminal ileum, where both illnesses frequently affect Peyer’s patches and solitary lymphoid follicles (Table 3).

**Table 3:** Clinical and pathological features comparing *Yersinia* infection and CD.

Feature	*Yersinia* infection	CD
Clinical presentation	• Acute, subacute, or chronic • Right lower quadrant pain (may mimic appendicitis) • Watery or bloody diarrhea • Fever (up to 40°C) • Nausea, vomiting (less common) • Extraintestinal: arthritis, skin lesions, sepsis, and mesenteric lymphadenopathy	• Chronic, relapsing IBD • Abdominal pain (often RLQ) • Diarrhea (may be bloody or mucus‐containing) • Weight loss, fatigue, and anemia • Extraintestinal: arthritis, eye/liver involvement, and skin lesions
Clinical course	• Acute, typically self‐limiting	• Chronic, relapsing‐remitting course
Onset	• Sudden onset • Incubation period: 3–7 days	• Gradual onset over weeks/months
Duration of infection	• Typically lasts 1–3 weeks	• Lifelong condition with periods of remission and relapse
Onset location	• Terminal ileum and mesenteric lymph nodes	• Any GI segment, commonly terminal ileum, colon, and rectum
Complications	• Dehydration, reactive arthritis, sepsis, and bowel perforation (rare)	• Fistulas, abscesses, strictures, and colorectal cancer risk
Imaging (CT/Sonography)	• Bowel wall thickening • Enlarged mesenteric lymph nodes	• Transmural inflammation • Bowel wall thickening • Inflamed, non‐compressible fat
Endoscopic appearance	• Mucosal inflammation • Ulcerations, sometimes pseudopolyps (terminal ileum)	• Segmental inflammation • Ulcerations, cobblestone pattern, and strictures
Histopathology	• Acute inflammation • Lymphoid hyperplasia • Occasionally, granulomas	• Chronic inflammation • Crypt abscesses • Non‐caseating granulomas
Risk factors	• Contaminated food/water • Contact with infected animals	• Genetic predisposition • Microbiome imbalance • Environmental and drug‐related factors
Diagnostic tests	• Stool culture, serology • Blood tests (CRP, ESR) • Imaging (CT, ultrasound) • Endoscopy with biopsy	• Endoscopy with biopsy • Blood tests (CRP, ESR, anemia) • Imaging (CT, MRI) • Fecal calprotectin
Treatment	• Antibiotics (e.g., ciprofloxacin, doxycycline) • Supportive care (hydration, pain relief)	• Anti‐inflammatory agents (mesalamine, corticosteroids) • Immunosuppressants, biologics • Surgery (in some cases)
Prognosis	• Good prognosis with treatment • Rare chronic complications	• Variable; requires long‐term management • Risk of complications over time

Abbreviations: CD, Crohn’s disease; CRP, C‐reactive protein; CT, computed tomography; ESR, erythrocyte sedimentation rate; GI, gastrointestinal; IBD, inflammatory bowel disease; MRI, magnetic resonance imaging; RLQ, right lower quadrant.

The management of *Y. enterocolitica* is reliant on the clinical presentation, which comprises the severity of the infection and the presence of extraintestinal symptoms [174]. Mild manifestations of the disease, characterized by transient diarrhea and abdominal pain, are typically self‐limiting and do not necessitate therapy [174]. Bacteremia accompanied by elevated fever may necessitate the administration of antipyretic agents, fluids, and broad‐spectrum antibiotics. Considering that *Y. enterocolitica* produces both A and B β‐lactamases, combination antibiotic therapy (pending antimicrobial susceptibility testing) may be considered (e.g., a fluoroquinolone with an aminoglycoside, a third‐generation cephalosporin with an aminoglycoside, or a fluoroquinolone with a third‐generation cephalosporin) [175]. In contrast, the management of yersiniosis caused by *Y. pseudotuberculosis* tends to be more straightforward and clinically favorable owing to the absence of β‐lactamase production by this bacterium. As a result, antimicrobial therapy with agents such as ampicillin, fluoroquinolones, tetracyclines, or aminoglycosides is reasonable [148], while surgical intervention is reserved for cases that are severe/complicated.

Antimicrobial resistance in pathogenic *Yersinia* species comprises both intrinsic and acquired mechanisms. Intrinsic resistance is largely mediated by the chromosomal β‐lactamases BlaA and BlaB, conferring natural resistance to ampicillin and several first‐generation β‐lactam antibiotics [176]. By contrast, acquired resistance results from chromosomal mutations or horizontal acquisition of resistance genes, reducing susceptibility to fluoroquinolones, tetracyclines, and extended‐spectrum cephalosporins [177, 178]. Although acquired resistance remains uncommon, continued surveillance is warranted because resistance determinants can disseminate through mobile genetic elements and the food chain [176]. Resistance to trimethoprim‐sulfamethoxazole, chloramphenicol, and nalidixic acid has been documented, with strains exhibiting multidrug resistance (MDR) characteristics, especially among biotype 1A and 4/O:3 isolates obtained from animal‐derived diets [179, 180]. Genomic studies have highlighted the importance of transposable elements and plasmids in the dissemination of resistance genes [181]. The Tn2670 transposon, integrated into the chromosomes of Swedish outbreak strains of *Y. enterocolitica*, harbored genes that confer resistance to chloramphenicol (*catA1*), streptomycin (*aadA1*), and sulfonamides (*sul1*), in addition to a mercury resistance module, highlighting the potential for horizontal gene transfer (HGT) and environmental persistence [179]. Likewise, *Y. pseudotuberculosis* has demonstrated the ability to acquire multidrug‐resistant plasmids, with reports of multidrug‐resistant strains emerging from various geographical regions [182, 183]. Molecular investigation of strains recovered in Russia and France identified the presence of *IncN* and *IncHI2* conjugative plasmids, which harbored resistance genes to six antibiotic classes, including aminoglycosides, β‐lactams, chloramphenicol, and tetracycline [184]. Besides conventional resistance, *Y. pseudotuberculosis* demonstrates antibiotic tolerance, especially toward doxycycline [185]. Research on gene expression has shown that the regulation of porin genes (e.g., *ompF*) and tRNA‐modifying genes (e.g., *tusB*) is crucial for bacterial survival under antibiotic pressure. The downregulation of *tusB* seems to facilitate doxycycline tolerance. In contrast, its overexpression increases the bacterium’s susceptibility, suggesting a complex interaction among resistance, tolerance, and gene regulation in the presence of bacteriostatic drugs [186]. Also, epidemiological data indicate that resistance patterns in *Y. enterocolitica* differ geographically and according to farming practices; a comprehensive European study involving over 1000 porcine 4/O:3 isolates revealed that resistance beyond ampicillin was rare in northern and eastern Europe but markedly elevated in areas with more intensive antimicrobial use for livestock, such as southern Europe [187]. These findings underscore the importance of judicious antibiotic use in agriculture to prevent the development of antibiotic resistance. Overall, growing genetic evidence highlights the adaptability of *Yersinia* spp. in acquiring resistance, underscoring the need for continuous monitoring to inform treatment and public health policies.

Moreover, dietary therapies such as exclusive enteral nutrition (EEN) and probiotic therapy may eradicate *Yersinia* and modify the gut microbiota, potentially mitigating CD symptoms [188]. EEN causes remission by initiating beneficial alterations in the gut microbiome. It fosters the proliferation of beneficial bacteria, such as Lachnospiraceae, and elevates short‐chain fatty acids (SCFAs), which confer protection against inflammation [188].

## 8. Probiotic‐Based Treatments in *Yersinia* Infection

Recent research suggests that certain probiotic strains can mitigate *Y. enterocolitica* infections by exhibiting antibacterial effects, modulating host immunological responses, and enhancing intestinal epithelial barrier function [189–191]. For instance, Bosák et al. [189] demonstrated that recombinant *E. coli* strains (EcH22, EcColinfant, Ec1127, and Ec1145) synthesizing colicin *F*_*Y*_ significantly suppressed the proliferation of pathogenic *Y. enterocolitica* in vitro and dysbiotic mice, indicating the potential use of colicin‐producing probiotics for therapeutic interventions. Frick et al. [190] identified many commensal bacteria, including *Bifidobacterium adolescent*is DSM 20086, that can inhibit *Y. enterocolitica*‐induced inflammatory signaling, specifically NF‐κB activation and IL‐8 generation, in HT‐29 epithelial cells. Wittmann et al. [191] confirmed that *B. adolescentis* Reuter 1963 (ATCC 15705) facilitates protection by stimulating plasmacytoid dendritic cells (pDCs) and regulatory T cells, which are essential for restricting the spread of *Y. enterocolitica*. In their murine model, mice pretreated with *B. adolescentis* exhibited less weight loss and reduced splenic bacterial counts post‐infection. The depletion of pDCs abolished this protective effect, highlighting their essential role in mediating the immune response. Jurado‐Gámez et al. [192, 193] established that both *Lactobacillus plantarum* ATCC 8014 and *Lactococcus lactis* ATCC 11454 suppressed the growth of *Y. pseudotuberculosis* (NCTC 8580) in co‐culture systems. They found bioactive peptides (e.g., VAL‐TIR‐VAL) in probiotic supernatants using HPLC, which are likely responsible for the observed antibacterial activity. These strains exhibited tolerance to low pH, bile salts, and increased temperatures, demonstrating significant survivability in gastrointestinal environments. Additionally, *L. lactis* secreting LcrV ameliorated 2,4,6‐trinitrobenzenesulfonic acid (TNBS)‐ and dextran sulfate sodium (DSS)‐induced colitis by promoting IL‐10 production through TLR2 signaling, highlighting its immunomodulatory potential in experimental IBD while retaining activity against *Yersinia* [194]. Collectively, these studies highlight the therapeutic potential of probiotic‐based strategies to suppress *Yersinia* spp. while modulating host immune responses relevant to intestinal inflammation. Frick et al. [195] demonstrated that *Lactobacillus fermentum* DSMZ 20052 alleviates *Y. enterocolitica*‐induced inflammation in HeLa 229 and HT‐29 cells by inhibiting IL‐8 release through the suppression of the Rac, p38 MAPK, and NF‐κB pathways. The anti‐inflammatory impact was associated with the released phospholipid, underscoring the immunomodulatory function of bacterial metabolites. In addition, Lavermicocca et al. [196] showed that *Lactobacillus paracasei* and *Lactobacillus plantarum* strains suppressed *Y. enterocolitica* proliferation and markedly diminished its urease activity in vitro. Urease is an essential virulence component that enhances *Y. enterocolitica*’s acid resistance and mucosal colonization; its inhibition indicates the capacity of these strains to counteract pathogen survival mechanisms while also facilitating gut acidification, which limits *Y. enterocolitica* persistence.

The *in vivo* efficacy of *L. plantarum* has been confirmed by De Montijo‐Prieto et al. [197], who illustrated that the kefir‐derived strain *L. plantarum* C4, when administered to mice, enhanced mucosal immune responses, facilitated rapid elimination of *Y. enterocolitica* from Peyer’s patches, and elevated secretory IgA levels—essential for pathogen neutralization at mucosal surfaces. Moreover, Shi et al. [198] demonstrated that *Levilactobacillus brevis* 23017 contributes to intestinal protection during *Y. enterocolitica* infection by preserving epithelial integrity (e.g., via tight junction stabilization), preventing villus destruction, and mitigating oxidative stress. The effects were mechanistically associated with the control of the p38 MAPK and NF‐κB signaling pathways, highlighting the strain’s multifaceted protective role in immune regulation, barrier preservation, and protection against antioxidants.

Zadernowska et al. [199] found that the addition of *Lactobacillus acidophilus* LA‐5 to blue cheese inhibited the growth of *Y. enterocolitica* at refrigerated temperatures, indicating an effective strategy to enhance food safety through probiotic‐enriched dairy products. The findings generally demonstrate the therapeutic efficacy of probiotics in managing *Y. enterocolitica* infections. Various probiotic strains, notably *B. adolescentis*, *L. fermentum*, *L. plantarum*, and *L. brevis*, have demonstrated efficacy in preclinical models through the production of antimicrobial peptides, modulation of host immunity, inhibition of inflammatory signaling, and preservation of epithelial integrity (Table S3). Consequently, additional clinical validation is necessary for incorporating these advantages into human medicinal applications.

## 9. Limitations and Future Perspectives

Despite accumulating evidence linking *Yersinia* to IBD, several important limitations remain. Most available studies rely on culture, targeted PCR, or serological assays, resulting in considerable methodological heterogeneity and limiting cross‐study comparisons. Furthermore, although shotgun metagenomic sequencing has substantially advanced the characterization of microbial dysbiosis in IBD, it has not reproducibly identified *Yersinia* spp. as enriched taxa across independent IBD cohorts [200, 201]. This observation suggests that *Yersinia* is unlikely to represent a consistent microbial signature of IBD and that, if involved, it may persist at low abundance or only under specific ecological or host genetic conditions. Future longitudinal studies integrating culture‐based approaches with targeted molecular detection, shotgun metagenomics, and host multi‐omics analyses are needed to define the contribution of *Yersinia* to IBD pathogenesis.

## 10. Concluding Remarks

The intricate relationship between *Yersinia* spp. and IBD, particularly CD, highlights a multifaceted environment in which microbial, genetic, immunological, and environmental factors converge to influence disease onset, progression, and treatment response. Rather than being regarded solely as incidental findings, *Y. enterocolitica* and *Y. pseudotuberculosis* have been investigated for their potential role in intestinal inflammation in genetically susceptible individuals. However, despite compelling epidemiological and mechanistic evidence, their precise contribution to IBD pathogenesis remains incompletely understood, and a definitive causal relationship has yet to be established. Their ability to break epithelial barriers, alter host immunity via various secretion systems and effector proteins, and exploit microbial dysbiosis supports their potential role within the ecological dysregulation model of IBD rather than establishing them as primary disease‐initiating pathogens. At the molecular level, the interactions between *Yersinia*‐associated virulence factors and host immunological sensors—particularly via inflammasome activation, pyroptotic cell death, and dysregulated cytokine cascades—demonstrate a complex bacterial strategy for immune control and persistence. In conjunction with host genetic variations in NOD2, ATG16L1, and associated pathways, these interactions may contribute to the chronic mucosal inflammation typical of CD.

The therapeutic management of *Yersinia* infections varies from supportive care to tailored antibiotic treatments, which are limited by the bacterium’s β‐lactamase profiles. Recent advancements in probiotic research—specifically concerning *B. adolescentis*, *L. fermentum*, *L. plantarum*, and *L. brevis*—present intriguing supplementary techniques. These commensals exhibit the ability to reduce the pathogen load, restore immune homeostasis, enhance epithelial integrity, and reduce inflammatory signals. Emerging evidence also suggests that selected engineered probiotic strains may exert immunomodulatory effects in experimental colitis models, although their therapeutic potential in IBD requires further validation. Future longitudinal studies integrating culture‐based approaches with targeted molecular detection, shotgun metagenomics, and host multi‐omics analyses are needed to determine whether *Yersinia* represents a transient enteric infection, persistent colonizer, or secondary consequence of intestinal inflammation in IBD. Such efforts may facilitate the development of precision diagnostics and microbiome‐informed therapeutic strategies for patients with IBD.

## Author Contributions

Fattaneh Sabzehali performed the literature review, carried out the visualization and wrote the initial manuscript draft. Verena Zerbato, Stefano Di Bella, Stefano Morabito, and Mohammad Reza Zali reviewed and edited the manuscript. Abbas Yadegar conceptualized, designed and supervised the study, acquired funding, contributed to visualization, and critically revised the manuscript.

## Funding

This study was financially supported by a research grant (no. RIGLD 1331, IR.SBMU.RIGLD.REC.1404.009) from the Research Institute for Gastroenterology and Liver Diseases, Shahid Beheshti University of Medical Sciences, Tehran, Iran.

## Disclosure

All authors read and approved the final version of the manuscript.

## Ethics Statement

The authors have nothing to report.

## Conflicts of Interest

The authors declare no conflicts of interest.

## Supporting Information

Additional supporting information can be found online in the Supporting Information section.

## Supporting information


**Supporting Information** The supporting data include two supporting tables providing additional information supporting this review. Table S1 summarizes the principal immune molecules involved in UC and CD, including their expression profiles, cellular contributors, and functional roles. Table S2 summarizes probiotic strains investigated against *Y. enterocolitica* and *Y. pseudotuberculosis*, including their effects, mechanisms of action, and experimental approaches.

## Data Availability

No new datasets were created or analyzed in the course of this study.

## References

[1] Huttenhower C. , Kostic A. D. , and Xavier R. J. , Inflammatory Bowel Disease as a Model for Translating the Microbiome, Immunity. (2014) 40, no. 6, 843–854, 10.1016/j.immuni.2014.05.013. 24950204 PMC4135443

[2] Khan I. A. , Nayak B. , and Markandey M. , et al.Differential Prevalence of Pathobionts and Host Gene Polymorphisms in Chronic Inflammatory Intestinal Diseases: Crohn’s Disease and Intestinal Tuberculosis, PLoS One. (2021) 16, no. 8, 10.1371/journal.pone.0256098, e0256098. 34407136 PMC8372915

[3] Wang R. , Li Z. , Liu S. , and Zhang D. , Global, Regional and National Burden of Inflammatory Bowel Disease in 204 Countries and Territories From 1990 to 2019: A Systematic Analysis Based on the Global Burden of Disease Study 2019, BMJ Open. (2023) 13, no. 3, 10.1136/bmjopen-2022-065186, e065186. PMC10069527 36977543

[4] Park S. H. , Update on the Epidemiology of Inflammatory Bowel Disease in Asia: Where Are We Now?, Intestinal Research. (2022) 20, no. 2, 159–164, 10.5217/ir.2021.00115. 35508952 PMC9081986

[5] de Souza H. S. P. , Fiocchi C. , and Iliopoulos D. , The IBD Interactome: An Integrated View of Aetiology, Pathogenesis and Therapy, Nature Reviews Gastroenterology & Hepatology. (2017) 14, no. 12, 739–749, 10.1038/nrgastro.2017.110. 28831186

[6] Guan Q. , A Comprehensive Review and Update on the Pathogenesis of Inflammatory Bowel Disease, Journal of Immunology Research. (2019) 2019, 10.1155/2019/7247238, 7247238. 31886308 PMC6914932

[7] Zhao M. , Chu J. , and Feng S. , et al.Immunological Mechanisms of Inflammatory Diseases Caused by Gut Microbiota Dysbiosis: A Review, Biomedicine & Pharmacotherapy. (2023) 164, 10.1016/j.biopha.2023.114985, 114985. 37311282

[8] Kiron V. , Hayes M. , and Avni D. , Inflammatory Bowel Disease—A Peek Into the Bacterial Community Shift and Algae-Based ‘Biotic’ Approach to Combat the Disease, Trends in Food Science & Technology. (2022) 129, 210–220, 10.1016/j.tifs.2022.09.012.

[9] Ohkusa T. , Okayasu I. , Ogihara T. , Morita K. , Ogawa M. , and Sato N. , Induction of Experimental Ulcerative Colitis by *Fusobacterium varium* Isolated From Colonic Mucosa of Patients With Ulcerative Colitis, Gut. (2003) 52, no. 1, 79–83, 10.1136/gut.52.1.79. 12477765 PMC1773498

[10] Santana P. T. , Rosas S. L. B. , Ribeiro B. E. , Marinho Y. , and de Souza H. S. P. , Dysbiosis in Inflammatory Bowel Disease: Pathogenic Role and Potential Therapeutic Targets, International Journal of Molecular Sciences. (2022) 23, no. 7, 10.3390/ijms23073464, 3464. 35408838 PMC8998182

[11] Belkaid Y. and Hand T. W. , Role of the Microbiota in Immunity and Inflammation, Cell. (2014) 157, no. 1, 121–141, 10.1016/j.cell.2014.03.011. 24679531 PMC4056765

[12] Caruso R. , Lo B. C. , and Núñez G. , Host–Microbiota Interactions in Inflammatory Bowel Disease, Nature Reviews Immunology. (2020) 20, no. 7, 411–426, 10.1038/s41577-019-0268-7. 32005980

[13] Franzosa E. A. , Sirota-Madi A. , and Avila-Pacheco J. , et al.Gut Microbiome Structure and Metabolic Activity in Inflammatory Bowel Disease, Nature Microbiology. (2019) 4, no. 2, 293–305, 10.1038/s41564-018-0306-4. PMC6342642 30531976

[14] Sartor R. B. , Microbial Influences in Inflammatory Bowel Diseases, Gastroenterology. (2008) 134, no. 2, 577–594, 10.1053/j.gastro.2007.11.059. 18242222

[15] Sheehan D. , Moran C. , and Shanahan F. , The Microbiota in Inflammatory Bowel Disease, Journal of Gastroenterology. (2015) 50, no. 5, 495–507, 10.1007/s00535-015-1064-1. 25808229

[16] Abdelhadi L. A. M. , Ibrahim N. A. , and Essa M. O. A. , et al.Adherent-Invasive *Escherichia coli* (AIEC) in Crohn’s Disease: A Bibliometric Analysis of 25 Years of Research (1999-2025), Microorganisms. (2026) 14, no. 6, 10.3390/microorganisms14061183, 1183. 42354808 PMC13304388

[17] Sokolovskaya O. M. , Uzunovic J. , and Peng Y. , et al.Dysbiosis-Associated Gut Bacterium *Ruminococcus gnavus* Varies at the Strain Level in Its Ability to Utilize Key Mucin Component Sialic Acid, Microbiology Spectrum. (2025) 13, no. 8, 10.1128/spectrum.03090-24, e03090-24. 40642981 PMC12323340

[18] Yang Y. , Li R. , and Zhong Q. , et al.In Situ Gut Microbiota Editing: Enhancing Therapeutic Efficacy for Bacterial Colitis by Compatible Oral Hydrogel Microspheres With Phages, Nature Communications. (2025) 16, no. 1, 10.1038/s41467-025-65498-1, 9785. PMC12808749 41198711

[19] Yu M. , Ding H. , and Gong S. , et al.Fungal Dysbiosis Facilitates Inflammatory Bowel Disease by Enhancing CD4+ T Cell Glutaminolysis, Frontiers in Cellular and Infection Microbiology. (2023) 13, 10.3389/fcimb.2023.1140757, 1140757. 37124046 PMC10140311

[20] Kondo T. , Kondo S. , and Nakayama-Imaohji H. , et al.Comparative Analysis of Mucosa-Associated and Luminal Gut Microbiota in Pediatric Ulcerative Colitis, International Journal of Molecular Sciences. (2025) 26, no. 21, 10.3390/ijms262110775, 10775. 41226812 PMC12610624

[21] Lamps L. W. , Madhusudhan K. T. , and Havens J. M. , et al.Pathogenic *Yersinia* DNA Is Detected in Bowel and Mesenteric Lymph Nodes From Patients With Crohn’s Disease, The American Journal of Surgical Pathology. (2003) 27, no. 2, 220–227, 10.1097/00000478-200302000-00011. 12548169

[22] Baut G. Le , O’Brien C. , and Pavli P. , et al.Prevalence of *Yersinia* Species in the Ileum of Crohn’s Disease Patients and Controls, Frontiers in Cellular and Infection Microbiology. (2018) 8, 10.3389/fcimb.2018.00336, 336. 30298122 PMC6160741

[23] Simovic I. , Hilmi I. , and Ng R. T. , et al. *ATG16L1* rs2241880/T300A Increases Susceptibility to Perianal Crohn’s Disease: An Updated Meta-Analysis on Inflammatory Bowel Disease Risk and Clinical Outcomes, United European Gastroenterology Journal. (2024) 12, no. 1, 103–121, 10.1002/ueg2.12477. 37837511 PMC10859713

[24] Petnicki-Ocwieja T. , Hrncir T. , and Liu Y.-J. , et al.Nod2 Is Required for the Regulation of Commensal Microbiota in the Intestine, Proceedings of the National Academy of Sciences of the United States of America. (2009) 106, no. 37, 15813–15818, 10.1073/pnas.0907722106. 19805227 PMC2747201

[25] Verbikova V. , Borilova G. , Babak V. , and Moravkova M. , Prevalence, Characterization and Antimicrobial Susceptibility of *Yersinia enterocolitica* and Other *Yersinia* Species Found in Fruits and Vegetables From the European Union, Food Control. (2018) 85, 161–167, 10.1016/j.foodcont.2017.08.038.

[26] European Food Safety Authority (EFSA) and European Centre for Disease Prevention and Control (ECDC) , The European Union One Health 2023 Zoonoses Report, EFSA Journal. (2024) 22, no. 12, 10.2903/j.efsa.2024.9106, e9106. 39659847 PMC11629028

[27] Špačková M. , Daniel O. , Klimešová P. , and Ileninová Z. , Overview of Basic Epidemiological Characteristics and Descriptive Analysis of the Incidence of Human Yersiniosis in the Czech Republic in 2018-2020, Epidemiologie, Mikrobiologie, Imunologie. (2022) 71, no. 1, 32–39. 35477268

[28] Fang X. , Kang L. , Qiu Y.-F. , Li Z.-S. , and Bai Y. , *Yersinia enterocolitica* in Crohn’s Disease, Frontiers in Cellular and Infection Microbiology. (2023) 13, 10.3389/fcimb.2023.1129996, 1129996. 36968108 PMC10031030

[29] Durack J. and Lynch S. V. , The Gut Microbiome: Relationships With Disease and Opportunities for Therapy, Journal of Experimental Medicine. (2019) 216, no. 1, 20–40, 10.1084/jem.20180448. 30322864 PMC6314516

[30] Leu S. B. , Shulman S. C. , and Steelman C. K. , et al.Pathogenic *Yersinia* DNA in Intestinal Specimens of Pediatric Patients With Crohn’s Disease, Fetal and Pediatric Pathology. (2013) 32, no. 5-6, 367–370, 10.3109/15513815.2013.768744. 23611062

[31] Parte A. C. , LPSN—List of Prokaryotic Names With Standing in Nomenclature, Nucleic Acids Research. (2013) 42, no. D1, D613–D616, 10.1093/nar/gkt1111. 24243842 PMC3965054

[32] Bennasar-Figueras A. , The Natural and Clinical History of Plague: From the Ancient Pandemics to Modern Insights, Microorganisms. (2024) 12, no. 1, 10.3390/microorganisms12010146, 146. 38257973 PMC10818976

[33] Kenyon J. J. , Cunneen M. M. , and Reeves P. R. , Genetics and Evolution of *Yersinia pseudotuberculosis* O-Specific Polysaccharides: A Novel Pattern of O-Antigen Diversity, FEMS Microbiology Reviews. (2017) 41, no. 2, 200–217, 10.1093/femsre/fux002. 28364730 PMC5399914

[34] Petsios S. , Fredriksson-Ahomaa M. , Sakkas H. , and Papadopoulou C. , Conventional and Molecular Methods Used in the Detection and Subtyping of *Yersinia enterocolitica* in Food, International Journal of Food Microbiology. (2016) 237, 55–72, 10.1016/j.ijfoodmicro.2016.08.015. 27543816

[35] Kaneko K. and Hashimoto N. , Occurrence of *Yersinia enterocolitica* in Wild Animals, Applied and Environmental Microbiology. (1981) 41, no. 3, 635–638, 10.1128/aem.41.3.635-638.1981. 7224628 PMC243751

[36] Bottone E. J. , *Yersinia enterocolitica*: The Charisma Continues, Clinical Microbiology Reviews. (1997) 10, no. 2, 257–276, 10.1128/CMR.10.2.257. 9105754 PMC172919

[37] Neubauer H. , Hensel A. , Aleksic S. , and Meyer H. , Identification of *Yersinia enterocolitica* Within the Genus *Yersinia* , Systematic and Applied Microbiology. (2000) 23, no. 1, 58–62, 10.1016/S0723-2020(00)80046-6. 10879979

[38] Shayegani M. , Morse D. , DeForge I. , Root T. , Parsons L. M. , and Maupin P. S. , Microbiology of a Major Foodborne Outbreak of Gastroenteritis Caused by *Yersinia enterocolitica* Serogroup O:8, Journal of Clinical Microbiology. (1983) 17, no. 1, 35–40, 10.1128/jcm.17.1.35-40.1983. 6826707 PMC272569

[39] European Food Safety Authority (EFSA) and European Centre for Disease Prevention and Control (ECDC) , The European Union Summary Report on Trends and Sources of Zoonoses, Zoonotic Agents and Food-Borne Outbreaks in 2017, EFSA Journal. (2018) 16, no. 12, 10.2903/j.efsa.2018.5500, e05500. 32625785 PMC7009540

[40] Eurosurveillance Editorial Team , ECDC Publishes the Annual Epidemiological Report 2012, Eurosurveillance. 18, no. 10, 2013–20418. 10.2807/ese.18.10.20418-en 23515064

[41] Syczyło K. , Platt-Samoraj A. , and Bancerz-Kisiel A. , et al.The Prevalence of *Yersinia enterocolitica* in Game Animals in Poland, PLoS One. (2018) 13, no. 3, 10.1371/journal.pone.0195136, e0195136. 29596492 PMC5875811

[42] European Centre for Disease Prevention and Control (ECDC) , Yersiniosis, 2024, ECDC.

[43] Rosner B. M. , Stark K. , and Werber D. , Epidemiology of Reported *Yersinia enterocolitica* Infections in Germany, 2001–2008, BMC Public Health. (2010) 10, no. 1, 10.1186/1471-2458-10-337, 337. 20546575 PMC2905328

[44] Fredriksson-Ahomaa M. , Sing A. , Enteropathogenic *Yersinia* spp.: Easily Misidentified Species, Zoonoses: Infections Affecting Humans and Animals, 2022, Springer International Publishing, 1–25.

[45] Fredriksson-Ahomaa M. , Lindström M. , and Korkeala H. , Juneja V. K. and Sofos J. N. , *Yersinia enterocolitica* and *Yersinia pseudotuberculosis* , Pathogens and Toxins in Foods: Challenges and Interventions, 2014, ASM Press, 164–180.

[46] Liang J. , Duan R. , and Xia S. , et al.Ecology and Geographic Distribution of *Yersinia enterocolitica* Among Livestock and Wildlife in China, Veterinary Microbiology. (2015) 178, no. 1-2, 125–131, 10.1016/j.vetmic.2015.05.006. 25987302

[47] Nuorti J. P. , Niskanen T. , and Hallanvuo S. , et al.A Widespread Outbreak of *Yersinia pseudotuberculosis* O:3 Infection From Iceberg Lettuce, The Journal of Infectious Diseases. (2004) 189, no. 5, 766–774, 10.1086/381766. 14976592

[48] Laukkanen R. , Martínez P. O. , Siekkinen K.-M. , Ranta J. , Maijala R. , and Korkeala H. , Transmission of *Yersinia pseudotuberculosis* in the Pork Production Chain From Farm to Slaughterhouse, Applied and Environmental Microbiology. (2008) 74, no. 17, 5444–5450, 10.1128/AEM.02664-07. 18641149 PMC2546633

[49] Sabina Y. , Rahman A. , Ray R. C. , and Montet D. , *Yersinia enterocolitica*: Mode of Transmission, Molecular Insights of Virulence, and Pathogenesis of Infection, Journal of Pathogens. (2011) 2011, 1–10, 10.4061/2011/429069. PMC3335483 22567333

[50] Van Kruiningen H. J. , Joossens M. , and Vermeire S. , et al.Environmental Factors in Familial Crohn’s Disease in Belgium, Inflammatory Bowel Diseases. (2005) 11, no. 4, 360–365, 10.1097/01.MIB.0000158536.31557.90. 15803025

[51] Yagüe-Muñoz A. , Arnedo-Pena A. , and Herrera-León S. , et al.Epidemiología Descriptiva De Infección Por *Yersinia enterocolitica* en Un Área De Alta Incidencia Durante 8 años, 2006-2013. Proyecto EDICS, Enfermedades Infecciosas y Microbiología Clínica. (2019) 37, no. 7, 441–447, 10.1016/j.eimc.2018.11.002. 30553619

[52] Mahapatro M. , Erkert L. , and Becker C. , Cytokine-Mediated Crosstalk Between Immune Cells and Epithelial Cells in the Gut, Cells. (2021) 10, no. 1, 10.3390/cells10010111, 111. 33435303 PMC7827439

[53] Rescigno M. , The Intestinal Epithelial Barrier in the Control of Homeostasis and Immunity, Trends in Immunology. (2011) 32, no. 6, 256–264, 10.1016/j.it.2011.04.003. 21565554

[54] Chelakkot C. , Ghim J. , and Ryu S. H. , Mechanisms Regulating Intestinal Barrier Integrity and Its Pathological Implications, Experimental & Molecular Medicine. (2018) 50, no. 8, 1–9, 10.1038/s12276-018-0126-x. PMC6095905 30115904

[55] Ahmad R. , Sorrell M. F. , Batra S. K. , Dhawan P. , and Singh A. B. , Gut Permeability and Mucosal Inflammation: Bad, Good or Context Dependent, Mucosal Immunology. (2017) 10, no. 2, 307–317, 10.1038/mi.2016.128. 28120842 PMC6171348

[56] Kamali Dolatabadi R. , Feizi A. , Halaji M. , Fazeli H. , and Adibi P. , The Prevalence of Adherent-Invasive *Escherichia coli* and Its Association With Inflammatory Bowel Diseases: A Systematic Review and Meta-Analysis, Frontiers in Medicine. (2021) 8, 10.3389/fmed.2021.730243, 730243. 34926490 PMC8678049

[57] Nielsen H. L. , Dalager-Pedersen M. , and Nielsen H. , Risk of Inflammatory Bowel Disease After *Campylobacter jejuni* and *Campylobacter concisus* Infection: A Population-Based Cohort Study, Scandinavian Journal of Gastroenterology. (2019) 54, no. 3, 265–272, 10.1080/00365521.2019.1578406. 30905214

[58] de Souza H. S. P. and Fiocchi C. , Immunopathogenesis of IBD: Current State of the Art, Nature Reviews Gastroenterology & Hepatology. (2016) 13, no. 1, 13–27, 10.1038/nrgastro.2015.186. 26627550

[59] Maloy K. J. and Powrie F. , Intestinal Homeostasis and Its Breakdown in Inflammatory Bowel Disease, Nature. (2011) 474, no. 7351, 298–306, 10.1038/nature10208. 21677746

[60] Schmitt H. , Neurath M. F. , and Atreya R. , Role of the IL23/IL17 Pathway in Crohn’s Disease, Frontiers in Immunology. (2021) 12, 10.3389/fimmu.2021.622934, 622934. 33859636 PMC8042267

[61] Nemeth Z. H. , Bogdanovski D. A. , Barratt-Stopper P. , Paglinco S. R. , Antonioli L. , and Rolandelli R. H. , Crohn’s Disease and Ulcerative Colitis Show Unique Cytokine Profiles, Cureus. (2017) 9, no. 4, 10.7759/cureus.1177, e1177. 28533995 PMC5438231

[62] Blaschitz C. and Raffatellu M. , Th17 Cytokines and the Gut Mucosal Barrier, Journal of Clinical Immunology. (2010) 30, no. 2, 196–203, 10.1007/s10875-010-9368-7. 20127275 PMC2842875

[63] Chauhan G. and Rieder F. , The Pathogenesis of Inflammatory Bowel Diseases, Surgical Clinics of North America. (2025) 105, no. 2, 201–215, 10.1016/j.suc.2024.10.008. 40015812 PMC11868724

[64] Xavier R. J. and Podolsky D. K. , Unravelling the Pathogenesis of Inflammatory Bowel Disease, Nature. (2007) 448, no. 7152, 427–434, 10.1038/nature06005. 17653185

[65] Kaplan M. H. , Th9 Cells: Differentiation and Disease, Immunological Reviews. (2013) 252, no. 1, 104–115, 10.1111/imr.12028. 23405898 PMC3982928

[66] Saebo A. , Vik E. , Lange O. J. , and Matuszkiewicz L. , Inflammatory Bowel Disease Associated With *Yersinia enterocolitica* O:3 Infection, European Journal of Internal Medicine. (2005) 16, no. 3, 176–182, 10.1016/j.ejim.2004.11.008. 15967332

[67] Jang J.-H. , Shin H. W. , Lee J. M. , Lee H.-W. , Kim E.-C. , and Park S. H. , An Overview of Pathogen Recognition Receptors for Innate Immunity in Dental Pulp, Mediators of Inflammation. (2015) 2015, no. 1, 10.1155/2015/794143, 794143. 26576076 PMC4630409

[68] Liu X. , Zhang Z. , and Ruan J. , et al.Inflammasome-Activated Gasdermin D Causes Pyroptosis by Forming Membrane Pores, Nature. (2016) 535, no. 7610, 153–158, 10.1038/nature18629. 27383986 PMC5539988

[69] Shi J. , Zhao Y. , and Wang Y. , et al.Inflammatory Caspases Are Innate Immune Receptors for Intracellular LPS, Nature. (2014) 514, no. 7521, 187–192, 10.1038/nature13683. 25119034

[70] Zhen Y. and Zhang H. , NLRP3 Inflammasome and Inflammatory Bowel Disease, Frontiers in Immunology. (2019) 10, 2019–2276, 10.3389/fimmu.2019.00276. 30873162 PMC6403142

[71] Guo H. , Callaway J. B. , and Ting J. P.-Y. , Inflammasomes: Mechanism of Action, Role in Disease, and Therapeutics, Nature Medicine. (2015) 21, no. 7, 677–687, 10.1038/nm.3893. PMC4519035 26121197

[72] Neurath M. F. , Targeting Immune Cell Circuits and Trafficking in Inflammatory Bowel Disease, Nature Immunology. (2019) 20, no. 8, 970–979, 10.1038/s41590-019-0415-0. 31235952

[73] Erhardt M. and Dersch P. , Regulatory Principles Governing *Salmonella* and *Yersinia* Virulence, Frontiers in Microbiology. (2015) 6, 10.3389/fmicb.2015.00949, 949. 26441883 PMC4563271

[74] Chen S. , Thompson K. M. , and Francis M. S. , Environmental Regulation of *Yersinia* Pathophysiology, Frontiers in Cellular and Infection Microbiology. (2016) 6, 10.3389/fcimb.2016.00025, 25. 26973818 PMC4773443

[75] Héchard T. , Lu L. , Edgren T. , Celestine C. , and Wang H. , YmoA Functions as a Molecular Stress Sensor in *Yersinia* , Communications Biology. (2025) 8, no. 1, 10.1038/s42003-025-07675-y, 225. 39948215 PMC11825884

[76] Tsolis R. M. , Young G. M. , Solnick J. V. , and Bäumler A. J. , From Bench to Bedside: Stealth of Enteroinvasive Pathogens, Nature Reviews Microbiology. (2008) 6, no. 12, 883–892, 10.1038/nrmicro2012. 18955984

[77] Dhar M. S. and Virdi J. S. , Strategies Used by *Yersinia enterocolitica* to Evade Killing by the Host: Thinking Beyond Yops, Microbes and Infection. (2014) 16, no. 2, 87–95, 10.1016/j.micinf.2013.11.002. 24246617

[78] De Koning-Ward T. F. and Robins-Browne R. M. , Contribution of Urease to Acid Tolerance in *Yersinia enterocolitica* , Infection and Immunity. (1995) 63, no. 10, 3790–3795, 10.1128/iai.63.10.3790-3795.1995. 7558281 PMC173532

[79] Dube P. , Sasakawa C. , Interaction of *Yersinia* With the Gut: Mechanisms of Pathogenesis and Immune Evasion, Molecular Mechanisms of Bacterial Infection via the Gut, 2009, 337, Springer, 61–91. 10.1007/978-3-642-01846-6_3 19812980

[80] Zeissig S. , Bürgel N. , and Günzel D. , et al.Changes in Expression and Distribution of Claudin 2, 5 and 8 Lead to Discontinuous Tight Junctions and Barrier Dysfunction in Active Crohn’s Disease, Gut. (2006) 56, no. 1, 61–72, 10.1136/gut.2006.094375. 16822808 PMC1856677

[81] Wehkamp J. , Salzman N. H. , and Porter E. , et al.Reduced Paneth Cell α-Defensins in Ileal Crohn’s Disease, Proceedings of the National Academy of Sciences of the United States of America. (2005) 102, no. 50, 18129–18134, 10.1073/pnas.0505256102. 16330776 PMC1306791

[82] Stappenbeck T. S. and McGovern D. P. B. , Paneth Cell Alterations in the Development and Phenotype of Crohn’s Disease, Gastroenterology. (2017) 152, no. 2, 322–326, 10.1053/j.gastro.2016.10.003. 27729212 PMC5209278

[83] Hering N. A. , Richter J. F. , and Krug S. M. , et al. *Yersinia enterocolitica* Induces Epithelial Barrier Dysfunction Through Regional Tight Junction Changes in Colonic HT-29/B6 Cell Monolayers, Laboratory Investigation. (2011) 91, no. 2, 310–324, 10.1038/labinvest.2010.180. 20956974

[84] Bohn E. , Sonnabend M. , Klein K. , and Autenrieth I. B. , Bacterial Adhesion and Host Cell Factors Leading to Effector Protein Injection by Type III Secretion System, International Journal of Medical Microbiology. (2019) 309, no. 5, 344–350, 10.1016/j.ijmm.2019.05.008. 31178419

[85] Aepfelbacher M. , Trasak C. , and Wilharm G. , et al.Characterization of YopT Effects on Rho GTPases in *Yersinia enterocolitica*-Infected Cells, Journal of Biological Chemistry. (2003) 278, no. 35, 33217–33223, 10.1074/jbc.M303349200. 12791693

[86] Andor A. , Trulzsch K. , and Essler M. , et al.YopE of *Yersinia*, a GAP for Rho GTPases, Selectively Modulates Rac-Dependent Actin Structures in Endothelial Cells, Cellular Microbiology. (2001) 3, no. 5, 301–310, 10.1046/j.1462-5822.2001.00114.x. 11298653

[87] Palace S. G. , Proulx M. K. , Szabady R. L. , and Goguen J. D. , Gain-of-Function Analysis Reveals Important Virulence Roles for the *Yersinia pestis* Type III Secretion System Effectors YopJ, YopT, and YpkA, Infection & Immunity. (2018) 86, no. 9, 10.1128/IAI.00318-18, e00318-18. 29891548 PMC6105907

[88] Blonska M. and Lin X. , NF-κB Signaling Pathways Regulated by CARMA Family of Scaffold Proteins, Cell Research. (2011) 21, no. 1, 55–70, 10.1038/cr.2010.182. 21187856 PMC3193407

[89] McCormick L. E. , Suarez C. , and Herring L. E. , et al.Multi-Monoubiquitylation Controls VASP-Mediated Actin Dynamics, Journal of Cell Science. (2024) 137, no. 2, 10.1242/jcs.261527, jcs261527. 38277158 PMC10917064

[90] Singaravelu P. , Lee W. L. , and Wee S. , et al. *Yersinia* Effector Protein (YopO)-Mediated Phosphorylation of Host Gelsolin Causes Calcium-Independent Activation Leading to Disruption of Actin Dynamics, Journal of Biological Chemistry. (2017) 292, no. 19, 8092–8100, 10.1074/jbc.M116.757971. 28280241 PMC5427284

[91] Logsdon L. K. and Mecsas J. , Requirement of the *Yersinia pseudotuberculosis* Effectors YopH and YopE in Colonization and Persistence in Intestinal and Lymph Tissues, Infection & Immunity. (2003) 71, no. 8, 4595–4607, 10.1128/IAI.71.8.4595-4607.2003. 12874339 PMC166012

[92] Pha K. , *Yersinia* Type III Effectors Perturb Host Innate Immune Responses, World Journal of Biological Chemistry. (2016) 7, no. 1, 1–13, 10.4331/wjbc.v7.i1.1. 26981193 PMC4768113

[93] Tseneva G. Y. , Solodovnikova N. Y. , and Voskresenskaya E. V. , Molecular Aspects of *Yersinia* Virulence, Microbiology and Antimicrobial Chemotherapy. (2002) 4, 248–266.

[94] Rolán H. G. , Durand E. A. , and Mecsas J. , Identifying *Yersinia* YopH-Targeted Signal Transduction Pathways That Impair Neutrophil Responses During *In Vivo*, Murine Infection, Cell Host & Microbe. (2013) 14, no. 3, 306–317, 10.1016/j.chom.2013.08.013. 24034616 PMC3789382

[95] Fasciano A. C. , Dasanayake G. S. , and Estes M. K. , et al. *Yersinia pseudotuberculosis* YopE Prevents Uptake by M Cells and Instigates M Cell Extrusion in Human Ileal Enteroid-Derived Monolayers, Gut Microbes. (2021) 13, no. 1, 10.1080/19490976.2021.1988390, 1988390. 34793276 PMC8604394

[96] Chaoprasid P. and Dersch P. , The Cytotoxic Necrotizing Factors (CNFs)—A Family of Rho GTPase-Activating Bacterial Exotoxins, Toxins. (2021) 13, no. 12, 10.3390/toxins13120901, 901. 34941738 PMC8709095

[97] Lockman H. A. , Gillespie R. A. , Baker B. D. , and Shakhnovich E. , *Yersinia pseudotuberculosis* Produces a Cytotoxic Necrotizing Factor, Infection & Immunity. (2002) 70, no. 5, 2708–2714, 10.1128/IAI.70.5.2708-2714.2002. 11953417 PMC127951

[98] Goubard A. , Loïez C. , and Abe J. , et al.Superantigenic *Yersinia pseudotuberculosis* Induces the Expression of Granzymes and Perforin by CD4+ T Cells, Infection & Immunity. (2015) 83, no. 5, 2053–2064, 10.1128/IAI.02339-14. 25754199 PMC4399040

[99] Sadana P. , Geyer R. , and Pezoldt J. , et al.The Invasin D Protein From *Yersinia pseudotuberculosis* Selectively Binds the Fab Region of Host Antibodies and Affects Colonization of the Intestine, Journal of Biological Chemistry. (2018) 293, no. 22, 8672–8690, 10.1074/jbc.RA117.001068. 29535184 PMC5986219

[100] von Tils D. , Blädel I. , Schmidt M. A. , and Heusipp G. , Type II Secretion in *Yersinia*—A Secretion System for Pathogenicity and Environmental Fitness, Frontiers in Cellular and Infection Microbiology. (2012) 2, 10.3389/fcimb.2012.00160, 160. 23248779 PMC3521999

[101] Iwobi A. , Heesemann J. , Garcia E. , Igwe E. , Noelting C. , and Rakin A. , Novel Virulence-Associated Type II Secretion System Unique to High-Pathogenicity *Yersinia enterocolitica* , Infection & Immunity. (2003) 71, no. 4, 1872–1879, 10.1128/IAI.71.4.1872-1879.2003. 12654803 PMC152056

[102] Cianfanelli F. R. , Monlezun L. , and Coulthurst S. J. , Aim, Load, Fire: The Type VI Secretion System, a Bacterial Nanoweapon, Trends in Microbiology. (2016) 24, no. 1, 51–62, 10.1016/j.tim.2015.10.005. 26549582

[103] Yang X. , Pan J. , Wang Y. , and Shen X. , Type VI Secretion Systems Present New Insights on Pathogenic *Yersinia* , Frontiers in Cellular and Infection Microbiology. (2018) 8, 10.3389/fcimb.2018.00260, 260. 30109217 PMC6079546

[104] Pessentheiner A. R. , Pelzmann H. J. , and Walenta E. , et al.NAT8L (N-Acetyltransferase 8-Like) Accelerates Lipid Turnover and Increases Energy Expenditure in Brown Adipocytes, Journal of Biological Chemistry. (2013) 288, no. 50, 36040–36051, 10.1074/jbc.M113.491324. 24155240 PMC3861652

[105] López-Pérez W. , Sai K. , Sakamachi Y. , Parsons C. , Kathariou S. , and Ninomiya-Tsuji J. , TAK1 Inhibition Elicits Mitochondrial ROS to Block Intracellular Bacterial Colonization, Proceedings of the National Academy of Sciences of the United States of America. (2021) 118, no. 25, 10.1073/pnas.2023647118, e2023647118. 34161265 PMC8237594

[106] Denecker G. , Declercq W. , and Geuijen C. A. W. , et al. *Yersinia enterocolitica* YopP-Induced Apoptosis of Macrophages Involves the Apoptotic Signaling Cascade Upstream of Bid, Journal of Biological Chemistry. (2001) 276, no. 23, 19706–19714, 10.1074/jbc.M101573200. 11279213

[107] Philip N. H. , Dillon C. P. , and Snyder A. G. , et al.Caspase-8 Mediates Caspase-1 Processing and Innate Immune Defense in Response to Bacterial Blockade of NF-κB and MAPK Signaling, Proceedings of the National Academy of Sciences of the United States of America. (2014) 111, no. 20, 7385–7390, 10.1073/pnas.1403252111. 24799700 PMC4034241

[108] Dubyak G. R. , Miller B. A. , and Pearlman E. , Pyroptosis in Neutrophils: Multimodal Integration of Inflammasome and Regulated Cell Death Signaling Pathways, Immunological Reviews. (2023) 314, no. 1, 229–249, 10.1111/imr.13186. 36656082 PMC10407921

[109] Chen K. W. , Demarco B. , and Ramos S. , et al.RIPK1 Activates Distinct Gasdermins in Macrophages and Neutrophils Upon Pathogen Blockade of Innate Immune Signaling, Proceedings of the National Academy of Sciences of the United States of America. (2021) 118, no. 28, 10.1073/pnas.2101189118, e2101189118. 34260403 PMC8285957

[110] Bergsbaken T. and Cookson B. T. , Macrophage Activation Redirects *Yersinia*-Infected Host Cell Death From Apoptosis to Caspase-1-Dependent Pyroptosis, PLoS Pathogens. (2007) 3, no. 11, 10.1371/journal.ppat.0030161, e161. 17983266 PMC2048529

[111] Sheppe A. E. F. , Santelices J. , Czyz D. M. , and Edelmann M. J. , *Yersinia pseudotuberculosis* YopJ Limits Macrophage Response by Downregulating COX-2-Mediated Biosynthesis of PGE2 in a MAPK/ERK-Dependent Manner, Microbiology Spectrum. (2021) 9, no. 1, 10.1128/Spectrum.00496-21, e00496-21. PMC8552654 34319170

[112] Seabaugh J. A. and Anderson D. M. , Pathogenicity and Virulence of *Yersinia* , Virulence. (2024) 15, no. 1, 10.1080/21505594.2024.2316439, 2316439. 38389313 PMC10896167

[113] Brodsky I. E. , Palm N. W. , and Sadanand S. , et al.A *Yersinia* Effector Protein Promotes Virulence by Preventing Inflammasome Recognition of the Type III Secretion System, Cell Host & Microbe. (2010) 7, no. 5, 376–387, 10.1016/j.chom.2010.04.009. 20478539 PMC2883865

[114] Thorslund S. E. , Edgren T. , and Pettersson J. , et al.The RACK1 Signaling Scaffold Protein Selectively Interacts With *Yersinia pseudotuberculosis* Virulence Function, PLoS One. (2011) 6, no. 2, 10.1371/journal.pone.0016784, e16784. 21347310 PMC3037380

[115] Miao E. A. , Leaf I. A. , and Treuting P. M. , et al.Caspase-1-Induced Pyroptosis Is an Innate Immune Effector Mechanism Against Intracellular Bacteria, Nature Immunology. (2010) 11, no. 12, 1136–1142, 10.1038/ni.1960. 21057511 PMC3058225

[116] Tai P. and Ascoli M. , Reactive Oxygen Species (ROS) Play a Critical Role in the cAMP-Induced Activation of Ras and the Phosphorylation of ERK1/2 in Leydig Cells, Molecular Endocrinology. (2011) 25, no. 5, 885–893, 10.1210/me.2010-0489. 21330403 PMC3386528

[117] Rana R. R. , Zhang M. , Spear A. M. , Atkins H. S. , and Byrne B. , Bacterial TIR-Containing Proteins and Host Innate Immune System Evasion, Medical Microbiology and Immunology. (2013) 202, no. 1, 1–10, 10.1007/s00430-012-0253-2. 22772799

[118] Coronas-Serna J. M. , Louche A. , and Rodríguez-Escudero M. , et al.The TIR-Domain-Containing Effectors BtpA and BtpB From *Brucella abortus* Impact NAD Metabolism, PLoS Pathogens. (2020) 16, no. 4, 10.1371/journal.ppat.1007979, e1007979. 32298382 PMC7188309

[119] Wan L. , TIR Enzymatic Functions: Signaling Molecules and Receptor Mechanisms, aBIOTECH. (2023) 4, no. 2, 172–175, 10.1007/s42994-023-00104-w. 37581018 PMC10423176

[120] He Y.-X. , Ye C.-L. , and Zhang P. , et al. *Yersinia pseudotuberculosis* Exploits CD209 Receptors for Promoting Host Dissemination and Infection, Infection & Immunity. (2019) 87, no. 1, 10.1128/IAI.00654-18, e00654-18. 30348825 PMC6300620

[121] Evans L. , Rhodes A. , and Alhazzani W. , et al.Surviving Sepsis Campaign: International Guidelines for Management of Sepsis and Septic Shock 2021, Intensive Care Medicine. (2021) 47, no. 11, 1181–1247, 10.1007/s00134-021-06506-y. 34599691 PMC8486643

[122] Wang Y. , Xiang Y. , and Lei C. , et al.Liver Abscess and Splenic Infarction Due to *Yersinia pseudotuberculosis* Bloodstream Infection: A Case Report, BMC Infectious Diseases. (2024) 24, 1415. 39695397 10.1186/s12879-024-10325-z PMC11654157

[123] Davicino R. C. , Méndez-Huergo S. P. , and Eliçabe R. J. , et al.Galectin-1-Driven Tolerogenic Programs Aggravate *Yersinia enterocolitica* Infection by Repressing Antibacterial Immunity, The Journal of Immunology. (2017) 199, no. 4, 1382–1392, 10.4049/jimmunol.1700579. 28716827

[124] Netea M. G. , van der Leij F. , and Drenth J. P. H. , et al.Chronic Yersiniosis Due to Defects in the TLR5 and NOD2 Recognition Pathways, The Netherlands Journal of Medicine. (2010) 68, no. 10, 310–315. 21071776

[125] Sugiura Y. , Kamdar K. , Khakpour S. , Young G. , Karpus W. J. , and DePaolo R. W. , TLR1-Induced Chemokine Production Is Critical for Mucosal Immunity Against *Yersinia enterocolitica* , Mucosal Immunology. (2013) 6, no. 6, 1101–1109, 10.1038/mi.2013.5. 23443468 PMC3760963

[126] Bancerz-Kisiel A. , Szczerba-Turek A. , Platt-Samoraj A. , and Szweda W. , Distribution of the *ymoA* and *ystA* Genes and Enterotoxin Yst Production by *Yersinia enterocolitica* Strains Isolated From Humans and Pigs, Polish Journal of Veterinary Sciences. (2012) 15, no. 4, 609–614, 10.2478/v10181-012-0096-1. 23390748

[127] Carniel E. , The High-Pathogenicity Island: An Iron-Uptake Island, Microbes and Infection. (2001) 3, no. 7, 561–569, 10.1016/S1286-4579(01)01412-5. 11418330

[128] Kappaun K. , Piovesan A. R. , Carlini C. R. , and Ligabue-Braun R. , Ureases: Historical Aspects, Catalytic, and Non-Catalytic Properties—A Review, Journal of Advanced Research. (2018) 13, 3–17, 10.1016/j.jare.2018.05.010. 30094078 PMC6077230

[129] Shutinoski B. , Schmidt M. A. , and Heusipp G. , Transcriptional Regulation of the Yts1 Type II Secretion System of *Yersinia enterocolitica* and Identification of Secretion Substrates, Molecular Microbiology. (2010) 75, no. 3, 676–691, 10.1111/j.1365-2958.2009.06998.x. 19968791

[130] Maldonado R. F. , Sá-Correia I. , and Valvano M. A. , Lipopolysaccharide Modification in Gram-Negative Bacteria During Chronic Infection, FEMS Microbiology Reviews. (2016) 40, no. 4, 480–493, 10.1093/femsre/fuw007. 27075488 PMC4931227

[131] Carnoy C. , Floquet S. , and Marceau M. , et al.The Superantigen Gene *ypm* Is Located in an Unstable Chromosomal Locus of *Yersinia pseudotuberculosis* , Journal of Bacteriology. (2002) 184, no. 16, 4489–4499, 10.1128/JB.184.16.4489-4499.2002. 12142419 PMC135243

[132] Homewood R. , Gibbons C. P. , Richards D. , Lewis A. , Duane P. D. , and Griffiths A. P. , Ileitis Due to *Yersinia pseudotuberculosis* in Crohn’s Disease, Journal of Infection. (2003) 47, no. 4, 328–332, 10.1016/S0163-4453(03)00064-1. 14556758

[133] Nörenberg D. , Wieser A. , and Magistro G. , et al.Molecular Analysis of a Novel Toll/Interleukin-1 Receptor (TIR)-Domain-Containing Virulence Protein of *Yersinia pseudotuberculosis* Among Far East Scarlet-Like Fever Serotype I Strains, International Journal of Medical Microbiology. (2013) 303, no. 8, 583–594, 10.1016/j.ijmm.2013.08.002. 24018301

[134] Schweer J. , Kulkarni D. , and Kochut A. , et al.The Cytotoxic Necrotizing Factor of *Yersinia pseudotuberculosis* (CNFY) Enhances Inflammation and Yop Delivery During Infection by Activation of Rho GTPases, PLoS Pathogens. (2013) 9, no. 11, 10.1371/journal.ppat.1003746, e1003746. 24244167 PMC3820761

[135] Bröms J. E. , Francis M. S. , and Forsberg Å. , Diminished LcrV Secretion Attenuates *Yersinia pseudotuberculosis* Virulence, Journal of Bacteriology. (2007) 189, no. 23, 8417–8429, 10.1128/JB.00936-07. 17873031 PMC2168923

[136] Viboud G. I. , Mejia E. , and Bliska J. B. , Comparison of YopE and YopT Activities in Counteracting Host Signaling Responses to *Yersinia pseudotuberculosis* Infection, Cellular Microbiology. (2006) 8, no. 9, 1504–1515, 10.1111/j.1462-5822.2006.00729.x. 16922868

[137] Adolph T. E. , Tomczak M. F. , and Niederreiter L. , et al.Paneth Cells as a Site of Origin for Intestinal Inflammation, Nature. (2013) 503, no. 7475, 272–276, 10.1038/nature12599. 24089213 PMC3862182

[138] Gardet A. and Xavier R. J. , Common Alleles That Influence Autophagy and the Risk for Inflammatory Bowel Disease, Current Opinion in Immunology. (2012) 24, no. 5, 522–529, 10.1016/j.coi.2012.08.001. 23041451

[139] Glas J. , Seiderer J. , and Tillack C. , et al.The *NOD2* Single Nucleotide Polymorphisms rs2066843 and rs2076756 Are Novel and Common Crohn’s Disease Susceptibility Gene Variants, PLoS One. (2010) 5, no. 12, 10.1371/journal.pone.0014466, e14466. 21209938 PMC3012690

[140] Corridoni D. , Kodani T. , and Rodriguez-Palacios A. , et al.Dysregulated *NOD2* Predisposes SAMP1/YitFc Mice to Chronic Intestinal Inflammation, Proceedings of the National Academy of Sciences of the United States of America. (2013) 110, no. 42, 16999–17004, 10.1073/pnas.1311657110. 24082103 PMC3801018

[141] Zheng D. , Liwinski T. , and Elinav E. , Interaction Between Microbiota and Immunity in Health and Disease, Cell Research. (2020) 30, no. 6, 492–506, 10.1038/s41422-020-0332-7. 32433595 PMC7264227

[142] Strober W. and Watanabe T. , *NOD2*, an Intracellular Innate Immune Sensor Involved in Host Defense and Crohn’s Disease, Mucosal Immunology. (2011) 4, no. 5, 484–495, 10.1038/mi.2011.29. 21750585 PMC3773501

[143] Hampe J. , Franke A. , and Rosenstiel P. , et al.A Genome-Wide Association Scan of Nonsynonymous SNPs Identifies a Susceptibility Variant for Crohn Disease in *ATG16L1* , Nature Genetics. (2007) 39, no. 2, 207–211, 10.1038/ng1954. 17200669

[144] Cadwell K. , Liu J. Y. , and Brown S. L. , et al.A Key Role for Autophagy and the Autophagy Gene *Atg16l1* in Mouse and Human Intestinal Paneth Cells, Nature. (2008) 456, no. 7219, 259–263, 10.1038/nature07416. 18849966 PMC2695978

[145] Rioux J. D. , Xavier R. J. , and Taylor K. D. , et al.Genome-Wide Association Study Identifies New Susceptibility Loci for Crohn Disease and Implicates Autophagy in Disease Pathogenesis, Nature Genetics. (2007) 39, no. 5, 596–604, 10.1038/ng2032. 17435756 PMC2757939

[146] Zhang H.-F. , Qiu L.-X. , and Chen Y. , et al. *ATG16L1* T300A Polymorphism and Crohn’s Disease Susceptibility: Evidence From 13,022 Cases and 17,532 Controls, Human Genetics. (2009) 125, no. 5-6, 627–631, 10.1007/s00439-009-0660-7. 19337756

[147] Kamdar K. , Khakpour S. , and Chen J. , et al.Genetic and Metabolic Signals During Acute Enteric Bacterial Infection Alter the Microbiota and Drive Progression to Chronic Inflammatory Disease, Cell Host & Microbe. (2016) 19, no. 1, 21–31, 10.1016/j.chom.2015.12.006. 26764594 PMC4839196

[148] Revés J. , Frias-Gomes C. , Ramos L. R. , and Glória L. , Positive *Yersinia* Serology and Colonic Cobblestone Pattern: A Diversion or Main Culprit?, GE—Portuguese Journal of Gastroenterology. (2025) 32, no. 2, 143–149, 10.1159/000541220. 40171098 PMC11961116

[149] Doshi P. , Sivaram G. , and Sievers C. , An Atypical Case of *Yersinia enterocolitica* Infection in a Patient Suspected With Ulcerative Colitis Flare-Up, Cureus. (2024) 16, no. 2, 10.7759/cureus.53780, e53780. 38465053 PMC10923545

[150] Filipiuk A. and Gonciarz M. , Rare Extraintestinal Manifestations of Ulcerative Colitis Treated With Dual Biologic Therapy: A Case Report, World Journal of Clinical Cases. (2024) 12, no. 23, 5441–5447, 10.12998/wjcc.v12.i23.5441. 39156084 PMC11238694

[151] Braga L. V. , Pedro W. J. S. , and Tabata K. S. I. , et al.Ileitis Due to *Yersinia* as a Differential Diagnosis of Crohn’s Disease: Case Report, Concilium. (2024) 24, no. 13, 591–596, 10.53660/CLM-3671-2441D.

[152] Marubashi K. , Takagi H. , and Wakagi T. , et al.Endoscopic and Video Capsule Endoscopic Observation of *Yersinia* Enterocolitis, DEN Open. (2023) 3, no. 1, 10.1002/deo2.242, e242. 37125071 PMC10140541

[153] Sood S. , Urquhart S. , and Coelho-Prabhu N. , S3180 *Yersiniosis*: An Overlooked Mimicker of Crohn’s Disease, American Journal of Gastroenterology. (2023) 118, no. 10S, 10.14309/01.ajg.0000962360.25579.c5, S2120.

[154] Ali M. Z. , Tariq M. U. , and Abid M. H. , et al.A Case Report and Literature Review of Rectosigmoid Crohn’s Disease: A Diagnostic Pitfall Ultimately Leading to Spontaneous Colonic Perforation, Cureus. (2023) 15, no. 4, 10.7759/cureus.36941, e36941. 37131553 PMC10148968

[155] Norrito R. L. , Pintus C. , and Cataldi M. , et al.A Case of Infective Colitis Due to *Yersinia enterocolitica* Complicated by Microliver Abscesses Mimicking Multiple Liver Occult Metastases: A Case Report, BMC Infectious Diseases. (2021) 21, no. 1, 10.1186/s12879-021-06177-6, 517. 34078290 PMC8173975

[156] Cian S. , Tabacco O. , and Costaguta A. , Terminal Ileitis Due to *Yersinia enterocolitica*: Differential Diagnosis With Crohn’s Disease in a 12-Year-Old Boy, Archivos Argentinos De Pediatría. (2020) 118, no. 2, e191–e194. 32199063 10.5546/aap.2020.e191

[157] Joung D. S. and Lee Y. M. , Terminal Ileitis Caused by *Yersinia enterocolitica* Presenting With Intussusception Mimicking Crohn’s Disease, Soonchunhyang Medical Science. (2019) 25, no. 2, 117–120, 10.15746/sms.19.022.

[158] Franczak P. , Witzling M. , and Siczewski W. , Yersiniosis or Leśniowski-Crohn’s Disease, Polish Journal of Surgery. (2018) 90, no. 1, 52–54, 10.5604/01.3001.0011.5966. 29513254

[159] Almeida C. M. G. , Maluf F. C. , Maluf F. C. , Lanzoni V. P. , Kiss D. R. , and Almeida M. G. , Crohn’s Disease Versus *Yersinia enterocolitica* Infection—Case Report: A Difficult Differential Diagnosis, Journal of Coloproctology. (2021) 38, no. 4, 343–345, 10.1016/j.jcol.2018.05.007.

[160] Wilczyńska D. , Mielniczuk K. , Szaflarska-Popławska A. , and Krogulska A. , Ulcerative Colitis in an Infant Aged 20 Months: A Case Report, Archivos Argentinos De Pediatría. (2018) 116, no. 6, e599–e603. 30016039 10.5546/aap.2018.eng.e599

[161] Naddei R. , Martinelli M. , and Strisciuglio C. , et al. *Yersinia enterocolitica* Ileitis Mimicking Pediatric Crohn’s Disease, Inflammatory Bowel Diseases. (2017) 23, no. 4, E15–E16, 10.1097/MIB.0000000000001052. 28230560

[162] Warner B. , Jayasooriya N. , Sanderson J. , Anderson S. , and Irving P. , *Yersinia*—A Rarer Cause of Terminal Ileitis, Archives of Case Reports in Clinical Medicine. (2016) 2, 123.

[163] Azghari I. , Bargach A. , Moatassim Billah N. , Essaoudi M. A. , Jahid A. , and Kabbaj N. , Ileocecal Resection for Massive Rectal Bleeding Due to *Yersinia enterocolitica*: A Case Report and Review of the Literature, Journal of Medical Case Reports. (2016) 10, no. 1, 10.1186/s13256-015-0786-2, 6. 26781434 PMC4717558

[164] Boţianu A. and Boţianu P. , A Case of *Yersinia enterocolitica* Diverticulitis Mimicking Inflammatory Bowel Disease: The Importance of Differential Diagnosis, Acta Medica Transilvanica. (2015) 20, no. 4, 1–3.

[165] Ijichi S. , Kusaka T. , Okada H. , Fujisawa T. , Kobara H. , and Itoh S. , Terminal Ileitis Caused by *Yersinia pseudotuberculosis* Mimicking Crohn Disease in Childhood, Journal of Pediatric Gastroenterology and Nutrition. (2012) 55, no. 4, e1–e3, 10.1097/MPG.0b013e318248a952. 22228001

[166] Safa G. , Loppin M. , Tisseau L. , and Lamoril J. , Cutaneous Aseptic Neutrophilic Abscesses and *Yersinia enterocolitica* Infection in a Case Subsequently Diagnosed as Crohn’s Disease, Dermatology. (2008) 217, no. 4, 340–342, 10.1159/000155646. 18799880

[167] Tuohy A. M. M. , O’Gorman M. , Byington C. , Reid B. , and Jackson W. D. , *Yersinia* Enterocolitis Mimicking Crohn’s Disease in a Toddler, Pediatrics. (1999) 104, no. 3, 10.1542/peds.104.3.e36, e36. 10469819

[168] Macfarlane P. I. and Miller V. , *Yersinia enterocolitica* Mimicking Crohn’s Disease, Journal of Pediatric Gastroenterology and Nutrition. (1986) 5, no. 4, 671–672, 10.1002/j.1536-4801.1986.tb09149.x. 3735020

[169] Lachman R. , Soong J. , Wishon G. , Maenza R. , Hanelin L. , and Geme J. S. , *Yersinia* Colitis, Gastrointestinal Radiology. (1977) 2, no. 1, 133–135, 10.1007/BF02256485. 615814

[170] Kallinowski F. , Wassmer A. , and Hofmann M. A. , et al.Prevalence of Enteropathogenic Bacteria in Surgically Treated Chronic Inflammatory Bowel Disease, Hepato-Gastroenterology. (1998) 45, no. 23, 1552–1558. 9840104

[171] Knösel T. , Schewe C. , Petersen N. , Dietel M. , and Petersen I. , Prevalence of Infectious Pathogens in Crohn’s Disease, Pathology–Research and Practice. (2009) 205, no. 4, 223–230, 10.1016/j.prp.2008.04.018. 19186006

[172] Ragnarsson E. G. E. , Schoultz I. , and Gullberg E. , et al. *Yersinia pseudotuberculosis* Induces Transcytosis of Nanoparticles Across Human Intestinal Villus Epithelium via Invasin-Dependent Macropinocytosis, Laboratory Investigation. (2008) 88, no. 11, 1215–1226, 10.1038/labinvest.2008.86. 18810251

[173] Chow J. , Lee S. M. , Shen Y. , Khosravi A. , and Mazmanian S. K. , Host–Bacterial Symbiosis in Health and Disease, Advances in Immunology, 2010, 107, Elsevier, 243–274. 21034976 10.1016/B978-0-12-381300-8.00008-3 PMC3152488

[174] Bockemühl J. and Roggentin P. , Enterale Yersiniosen: Klinische Bedeutung, Epidemiologie, Diagnostik Und Prävention, Bundesgesundheitsblatt–Gesundheitsforschung–Gesundheitsschutz. (2004) 47, no. 7, 732–739, 10.1007/s00103-004-0865-9. 15254824

[175] Lee Y. J. , Kim J. , and Jeon J. H. , et al.Extraintestinal Manifestation of *Yersinia pseudotuberculosis* Bacteremia as Acute Hepatitis: Case Report and Review of the Literature, Pathogens. (2021) 10, no. 10, 1255. 34684204 10.3390/pathogens10101255 PMC8539584

[176] Frazão M. R. , Andrade L. N. , Darini A. L. C. , and Falcão J. P. , Antimicrobial Resistance and Plasmid Replicons in *Yersinia enterocolitica* Strains Isolated in Brazil in 30 Years, The Brazilian Journal of Infectious Diseases. (2017) 21, no. 4, 477–480, 10.1016/j.bjid.2017.04.006. 28558260 PMC9427965

[177] Seakamela E. M. , Diseko L. , and Malatji D. , et al.Characterisation and Antibiotic Resistance of *Yersinia enterocolitica* From Various Meat Categories, South Africa, Onderstepoort Journal of Veterinary Research. (2022) 89, no. 1, e1–e8, 10.4102/ojvr.v89i1.2006. PMC9724029 36453823

[178] Sotohy S. A. , Diab M. S. , Ewida R. M. , Aballah A. , and Eldin N. K. A. , An Investigative Study on *Yersinia enterocolitica* in Animals, Humans and Dried Milk in New Valley Governorate, Egypt, BMC Microbiology. (2024) 24, no. 1, 10.1186/s12866-024-03527-7, 395. 39379816 PMC11462700

[179] Karlsson P. A. , Tano E. , and Jernberg C. , et al.Molecular Characterization of Multidrug-Resistant *Yersinia enterocolitica* From Foodborne Outbreaks in Sweden, Frontiers in Microbiology. (2021) 12, 10.3389/fmicb.2021.664665, 664665. 34054769 PMC8155512

[180] Ye Q. , Wu Q. , Hu H. , Zhang J. , and Huang H. , Prevalence, Antimicrobial Resistance and Genetic Diversity of *Yersinia enterocolitica* Isolated From Retail Frozen Foods in China, FEMS Microbiology Letters. (2015) 362, no. 24, 10.1093/femsle/fnv197, fnv197. 26472688

[181] Koren S. and Phillippy A. M. , One Chromosome, One Contig: Complete Microbial Genomes From Long-Read Sequencing and Assembly, Current Opinion in Microbiology. (2015) 23, 110–120, 10.1016/j.mib.2014.11.014. 25461581

[182] Gridnev V. A. , Dziubak V. F. , Zagoruĭko V. G. , Gridneva L. G. , and Zaĭnulina Z. U. , Conjugative R-Plasmid Resistance of the Causative Agents of Yersinia Infection, Antibiotiki i Khimioterapiya. (1990) 35, no. 5, 19–22. 2337369

[183] Tsubokura M. , Otsuki K. , and Sato K. , et al.Special Features of Distribution of *Yersinia pseudotuberculosis* in Japan, Journal of Clinical Microbiology. (1989) 27, no. 4, 790–791, 10.1128/jcm.27.4.790-791.1989. 2656752 PMC267423

[184] Cabanel N. , Galimand M. , Bouchier C. , Chesnokova M. , Klimov V. , and Carniel E. , Molecular Bases for Multidrug Resistance in *Yersinia pseudotuberculosis* , International Journal of Medical Microbiology. (2017) 307, no. 7, 371–381, 10.1016/j.ijmm.2017.08.005. 28830739

[185] Raneses J. R. , Ellison A. L. , Liu B. , and Davis K. M. , Subpopulations of Stressed *Yersinia pseudotuberculosis* Preferentially Survive Doxycycline Treatment Within Host Tissues, mBio. (2020) 11, no. 4, 10.1128/mBio.00901-20, e00901-20. 32753491 PMC7407081

[186] Alvarez-Manzo H. S. , Davidson R. K. , and Van Cauwelaert De Wyels J. , et al. *Yersinia pseudotuberculosis* Doxycycline Tolerance Strategies Include Modulating Expression of Genes Involved in Cell Permeability and tRNA Modifications, PLoS Pathogens. (2022) 18, no. 5, 10.1371/journal.ppat.1010556, e1010556. 35576231 PMC9135342

[187] Koskinen J. , Ortiz-Martínez P. , Keto-Timonen R. , Joutsen S. , Fredriksson-Ahomaa M. , and Korkeala H. , Prudent Antimicrobial Use Is Essential to Prevent the Emergence of Antimicrobial Resistance in *Yersinia enterocolitica* 4/O:3 Strains in Pigs, Frontiers in Microbiology. (2022) 13, 10.3389/fmicb.2022.841841, 841841. 35369517 PMC8967395

[188] Häcker D. , Siebert K. , and Smith B. J. , et al.Exclusive Enteral Nutrition Initiates Individual Protective Microbiome Changes to Induce Remission in Pediatric Crohn’s Disease, Cell Host & Microbe. (2024) 32, no. 11, 2019–2034.e8, 10.1016/j.chom.2024.10.001. 39461337 PMC12017801

[189] Bosák J. , Micenková L. , and Hrala M. , et al.Colicin FY Inhibits Pathogenic *Yersinia enterocolitica* in Mice, Scientific Reports. (2018) 8, no. 1, 10.1038/s41598-018-30729-7, 12242. 30115964 PMC6095899

[190] Frick J. S. , Fink K. , and Kahl F. , et al.Identification of Commensal Bacterial Strains That Modulate *Yersinia enterocolitica* and Dextran Sodium Sulfate-Induced Inflammatory Responses: Implications for the Development of Probiotics, Infection and Immunity. (2007) 75, no. 7, 3490–3497, 10.1128/IAI.00119-07. 17485456 PMC1932957

[191] Wittmann A. , Autenrieth I. B. , and Frick J.-S. , Plasmacytoid Dendritic Cells Are Crucial in *Bifidobacterium adolescentis*-Mediated Inhibition of *Yersinia enterocolitica* Infection, PLoS One. (2013) 8, no. 8, 10.1371/journal.pone.0071338, e71338. 23977019 PMC3748105

[192] Jurado Gámez H. , Romero Benavides A. , and Morillo Garces A. , Cinética, Prueba De Crecimiento y Efecto De Inhibición De *Lactococcus lactis* Sobre *Yersinia pseudotuberculosis* , Biotecnología en El Sector Agropecuario y Agroindustrial. (2016) 14, no. 2, 18–28, 10.18684/BSAA(14)18-28.

[193] Jurado-Gámez H. , Martínez B. J. A. , Chaspuengal T. A. , and Calpa Y. F. Y. , Evaluación *In Vitro* de la De LA Acción De *Lactobacillus plantarum* Con Características Probióticas Sobre *Yersinia pseudotuberculosis* , Biotecnología en El Sector Agropecuario y Agroindustrial. (2014) 12, no. 2, 49–59.

[194] Foligne B. , Dessein R. , and Marceau M. , et al.Prevention and Treatment of Colitis With *Lactococcus lactis* Secreting the Immunomodulatory *Yersinia* LcrV Protein, Gastroenterology. (2007) 133, no. 3, 862–874, 10.1053/j.gastro.2007.06.018. 17678918

[195] Frick J.-S. , Schenk K. , and Quitadamo M. , et al. *Lactobacillus fermentum* Attenuates the Proinflammatory Effect of *Yersinia enterocolitica* on Human Epithelial Cells, Inflammatory Bowel Diseases. (2007) 13, no. 1, 83–90, 10.1002/ibd.20009. 17206643

[196] Lavermicocca P. , Valerio F. , Lonigro S. L. , Di Leo A. , and Visconti A. , Antagonistic Activity of Potential Probiotic *Lactobacilli* Against the Ureolytic Pathogen *Yersinia enterocolitica* , Current Microbiology. (2008) 56, no. 2, 175–181, 10.1007/s00284-007-9069-5. 18074177

[197] De Montijo-Prieto S. , Moreno E. , and Bergillos-Meca T. , et al.A *Lactobacillus plantarum* Strain Isolated From Kefir Protects Against Intestinal Infection With *Yersinia enterocolitica* O:9 and Modulates Immunity in Mice, Research in Microbiology. (2015) 166, no. 8, 626–632, 10.1016/j.resmic.2015.07.010. 26272025

[198] Shi Z. , Guan N. , and Sun W. , et al.Protective Effect of *Levilactobacillus Brevis* Against *Yersinia enterocolitica* Infection in a Mouse Model via Regulating the MAPK and NF-κB Pathway, Probiotics and Antimicrobial Proteins. (2022) 14, no. 5, 830–844, 10.1007/s12602-022-09957-x. 35665480

[199] Zadernowska A. , Chajęcka-Wierzchowska W. , and Ogryzek M. P. , Growth Potential of *Yersinia enterocolitica* in Blue Cheese and Blue Cheese With Probiotic *Lactobacillus acidophilus* LA-5, Journal of Food Science and Technology. (2015) 52, no. 11, 7540–7544, 10.1007/s13197-015-1873-5.

[200] Aldars-García L. , Chaparro M. , and Gisbert J. P. , Systematic Review: The Gut Microbiome and Its Potential Clinical Application in Inflammatory Bowel Disease, Microorganisms. (2021) 9, no. 5, 10.3390/microorganisms9050977, 977. 33946482 PMC8147118

[201] Lloyd-Price J. , Arze C. , and Ananthakrishnan A. N. , et al.Multi-Omics of the Gut Microbial Ecosystem in Inflammatory Bowel Diseases, Nature. (2019) 569, no. 7758, 655–662, 10.1038/s41586-019-1237-9. 31142855 PMC6650278

