# Supplementary material for: Unraveling the Pathogenetic Potential and Immune Crosstalk of Yersinia spp. in Inflammatory Bowel Disease
Source: J Immunol Res. 2026 Aug 28;2026:2846665. doi: 10.1155/jimr/2846665 (PMC13522838; doi:10.1155/jimr/2846665)
Supplement: Supplementary file 1 [file JIMR-2026-2846665-s001.docx]

**TABLE S1:** Principal immune molecules and their expression profiles, cellular participants, and functional roles in UC and CD.

| **Immune component** | **Expression/production in UC and/or CD** | **Cellular contributors** | **Effects** | **Refs.** |
| --- | --- | --- | --- | --- |
| Integrin | | | | |
| α4β1 | - Increased expression | - T cells - Monocytes - Eosinophils | - Facilitates leukocyte recruitment to inflamed gut mucosa - Involved in alternative mechanism for recruiting inflammatory T cells | [1–3] |
| α4β7 | - Increased expression | - T cells - B cells | - Crucial for lymphocyte homing to the gut mucosa - Involved in intestinal recruitment of B cells/ASC |  |
| αEβ7 | - Increased expression | - Intraepithelial lymphocytes | - Involved in IEL adhesion to epithelial cells - May be upregulated by TGF-β in the gut mucosa |  |
| αLβ2 | - Increased expression | - T cells - B cells - Neutrophils - Monocytes - Macrophages | - Involved in leukocyte adhesion and migration to inflamed sites |  |
| **Proinflammatory cytokines** | | | | |
| IL-1α, IL-1β | - Increased | - Macrophages - Monocytes - Epithelial cells - Dendritic cells | - Inflammation initiation and amplification - Tissue damage - Epithelial cell apoptosis - Barrier dysfunction - Fibrosis - Regulation of Th17 immunity | [4,5] |
| IL-8 | - Altered in UC and CD - Increased in chronic ileal lesions of CD | - Macrophages - Endothelial cells - Leukocytes - Smooth muscle cells | - Drives inflammation, neutrophil activation, chemotaxis, contributes to Th17-related responses | [1] |
| IL-17 | - High levels in UC and CD tissues - Elevated serum levels in children with IBD | - Th17 cells - ILC3s | - Proinflammatory: induces neutrophil recruitment and activation, contributes to intestinal fibrosis, may play a role in abnormal humoral immunity   - Anti-microbial: enhances secretion of IgA and antimicrobial peptides, inhibits colonization of pathogenic bacteria - Homeostatic: maintains mucosal homeostasis (ILC3s) | [6] |
| IL-18 | - Increased | - Macrophages - Dendritic cells - Epithelial cells | - Proinflammatory: promotes Th1 cell differentiation, triggers IFN-γ production in Th1 cells and NK cells, contributes to breakdown of mucosal barriers - Anti-inflammatory: observed in early stages of DSS-induced colitis | [7] |
| IL-12/23 | - Overproduced | - Macrophages - Dendritic cells - T cells | - Proinflammatory: promotes Th1 cell differentiation, IFN-γ production, inflammation - Anti-inflammatory: involved in epithelial barrier integrity and defense against pathogens, contributes to chronic inflammation in CD and UC | [8,9] |
| IL-33 | - Increased in inflamed lesions | - Epithelial cells - Macrophages - Mast cells | Dual role:   - Can promote Th2 responses and inflammation - Can also contribute to mucosal regeneration and resolution of inflammation - Involved in fibrosis - Plays a role in maintaining intestinal homeostasis | [10,11] |
| IL-36 | - Overexpressed in both UC and CD | - Keratinocytes - Epithelial cells - Inflammatory monocytes/macrophages | - Pathogenic roles (inflammatory responses) - Protective roles (wound healing, resolution of inflammation) - Contributes to fibrosis in IBD - Promotes IL-22 expression | [12,13] |
| IL-38 | - Higher expression in active UC compared to active CD - Increased expression in inflamed tissue of both UC and CD compared to non-inflamed tissue | - Primarily B cells in inflamed mucosa of UC - Expressed in intestinal epithelial cells, macrophages, CD8^+^ T cells, and/or pDCs in active IBD | - Inhibits IL-17 and IL-22 - Deficiency associated with increased disease activity, weight loss, histological damage, and intestinal permeability | [13] |
| **Anti-inflammatory cytokines** | | | | |
| IL-4 | - Decreased in UC | - T cells - Mast cells - Eosinophils - Basophils | - Suppresses TNF-α, IL-6, and IL-1β - Promotes epithelial wound repair - Regulates antibody class-switching to IgE - May have a role in disease susceptibility based on polymorphisms | [14] |
| IL-6 | - Increased in both UC and CD | - Macrophages - T cells - B cells - Fibroblasts - Epithelial cells | - Proinflammatory cytokine - Contributes to inflammation and tissue damage - Promotes Th17 cell differentiation - Involved in CRP production - Associated with disease activity and severity | [8,15] |
| IL-9 | - High expression in UC, especially in severe cases | - Th9 cells (primarily) - Mast cells - Eosinophils | - Can be both proinflammatory and anti-inflammatory depending on the context - In UC: promotes inflammation by impairing barrier function (increased permeability), may contribute to mucosal damage - In CD: may have a protective role in some cases - Overall: contributes to T-cell activation in experimental colitis models | [16] |
| IL-10 | - Decreased in active disease - Polymorphisms associated with increased risk of UC, especially early onset - Mutations in IL-10, IL-10RA, and IL-10RB genes associated with severe early-onset IBD | - Macrophages - Dendritic cells - Neutrophils - NK cells - B cells - CD8^+^ T cells (primarily CD4^+^ T cells) | - Anti-inflammatory, suppresses proinflammatory responses, maintains intestinal homeostasis, limits tissue damage, inhibits Th17 cell responses | [17–19] |
| IL-11 | - Decreased expression and a failure to downregulate NF-κB | - Mesenchymal cells - Megakaryocytes - Epithelial cells | - Decreased expression, impaired NF-κB downregulation, potential role in UC pathogenesis | [20] |
| IL-13 | - Overexpressed in fibrotic areas of CD | - T cells (Th2) - Mast cells - Eosinophils | - Promotes tissue remodeling (crypt stemness, goblet cell hyperplasia) - Induces epithelial-to-mesenchymal transition - Upregulates matrix metallo-proteinases (leading to fistula formation) - Disrupts intestinal barrier integrity (upregulates claudin-2, promotes apoptosis) - Profibrotic activity (elevated TGF-β, stimulates myofibroblast differentiation, enhancing collagen production) | [21] |
| IL-22 | - Increased in active lesions of CD | - Th-17 cells - γδ T cells - ILCs | - Protective: maintains intestinal epithelial homeostasis and barrier function, promotes mucosal healing (epithelial cell regeneration, wound healing, mucus production), enhances antimicrobial immunity - Potentially detrimental: can promote inflammation in certain contexts, may contribute to tumor growth (in preclinical models), can downregulate goblet cells and secreted mucin-2 | [22–26] |
| **TNF-α signaling** | | | | |
| TNF-α | - Increased | - T cells - Macrophages - Monocytes | - Promotes inflammation, cell death, and tissue damage, contributes to disease activity and severity | [27] |
| TNFR1 | - Widely expressed | - Various cells | - Primary mediator of TNF-α's cytotoxic effects, involved in inflammation and tissue injury | [28] |
| TNFR2 | - Expressed on T cells | - T cells | - Promotes Th17 differentiation and enhances Th1 cytokine production, exacerbating inflammation | [29] |
| TNFAIP8L2 (TIPE2) | - Likely decreased | - CD4^+^ T cells - Dendritic cells - Other immune cells | - Downregulated in IBD, contributing to inflammation | [30,31] |
| TL1A (TNF-like cytokine 1A) | - Significantly upregulated | - Activated lymphocytes - T-reg cells | - Induces costimulatory signals to activated lymphocytes - Impacts Th1, Th2, and Th17 effector pathways - Contributes to mucosal inflammation and fibrosis - Associated with chronic inflammation and extra-intestinal manifestations | [32] |

*Note:* ASC, Antibody-secreting cells; CD4+ T cells, Cluster of differentiation 4 positive T cells; CD8+ T cells, Cluster of differentiation 8 positive T cells; IEL, Intraepithelial lymphocytes; IL-1α, Interleukin-1 alpha; IL-1β, Interleukin-1 beta; IL-4, Interleukin-4; ILC3s, Group 3 innate lymphoid cells; ILCs, Innate lymphoid cells; NK cells, Natural killer cells; pDCs, Plasmacytoid dendritic cells; TGF-β, Transforming growth factor beta; Th1, T helper type 1 cell; Th17, T helper type 17 cell; Th2, T helper type 2 cell; Th9, T helper type 9 cell; TL1A, TNF-like cytokine 1A; TNFAIP8L2 (TIPE2), Tumor necrosis factor alpha-induced protein 8-like 2 (also known as TIPE2); TNFR1, Tumor necrosis factor receptor 1; TNFR2, Tumor necrosis factor receptor 2; TNF-α, Tumor necrosis factor alpha; α4β1, Alpha-4 beta-1 integrin; α4β7, Alpha-4 beta-7 integrin; αEβ7, Alpha-E beta-7 integrin; αLβ2, Alpha-L beta-2 integrin.

**TABLE S2:** Major type III secretion system (T3SS) effector proteins (Yops) of pathogenic *Yersinia* and their roles in host immune modulation.

| **Yop effector** | **Primary target/mechanism** | **Major immunological effects** | **Potential implication in IBD** |
| --- | --- | --- | --- |
| **YopE** | Rho GTPases (RhoA, Rac1, Cdc42) | - Disrupts actin cytoskeleton - Inhibits phagocytosis and ROS production - Modulates inflammasome signaling and IL-1β responses | - May facilitate immune evasion - May prolong intestinal inflammation |
| **YopT** | Rho GTPases | - Inactivates Rho GTPases - Disrupts cytoskeletal organization - Impairs phagocytosis | - May promote bacterial persistence - May contribute to chronic mucosal inflammation |
| **YopO (YpkA)** | Rho GTPases (Rac1, RhoA) | - Disrupts cytoskeletal organization - Impairs phagocytosis - Suppresses neutrophil function - Promotes macrophage apoptosis | - Weakens innate immune defenses |
| **YopH** | Tyrosine-phosphorylated signaling proteins | - Inhibits phagocytosis - Suppresses calcium signaling - Reduces leukocyte activation and ROS production | - Suppresses antibacterial immune responses - Impairs bacterial clearance |
| **YopP (YopJ)** | MAPK, TAK1, and NF-κB signaling pathways | - Suppresses proinflammatory cytokine production - Induces macrophage apoptosis - Modulates pyroptosis - Promotes IL-1β release through RIPK1–caspase-8/GSDME signaling | - Alters intestinal immune homeostasis - Modulates inflammatory signaling |
| **YopM** | PRK2, RSK1, and pyrin inflammasome | - Suppresses pyrin inflammasome activation - Limits inflammatory cell death - Reduces neutrophil recruitment - Promotes anti-inflammatory responses | - May contribute to immune evasion - May impair bacterial clearance - May promote dysregulated intestinal inflammation |
| **YopQ (YopK in *Y. pseudotuberculosis*)** | - T3SS translocon - Inflammasome sensing | - Regulates Yop translocation - Limits inflammasome activation - Reduces neutrophil-mediated phagocytosis | - Promotes bacterial persistence - Sustains host–pathogen interactions |

ROS, reactive oxygen species; MAPK, mitogen-activated protein kinase; NF-κB, nuclear factor κB; T3SS, type III secretion system. Information summarized from the references cited in Section 4.2.

**TABLE S3:** Characterization of probiotic strains with therapeutic relevance to Yersinia-associated intestinal inflammation and IBD, including their effects, mechanisms of action, and experimental settings.

| **Probiotic strain** | **Cell line** | **Effects** | **Mechanism of action** | **Experimental approaches** | **Refs.** |
| --- | --- | --- | --- | --- | --- |
| *E. coli* strains producing colicin F_Y_ (EcH22, EcColinfant, Ec1127, Ec1145) | Not applicable | - Inhibits the growth and infection of pathogenic *Y. enterocolitica* *in vitro* and *in vivo* | - Production of colicin F_Y_, a bacteriocin that binds the *Yersinia*-specific outer membrane protein YiuR, leading to cell membrane pore formation and bacterial death | - Construction of recombinant *E. coli* strains expressing colicin F_Y_ using plasmids with *lac* and *pcib* promoters - Isolation and identification of murine *E. coli* strains from mouse feces (ENTEROtest, PFGE) - Growth in TY media and selective plating on CIN, kanamycin, chloramphenicol, and streptomycin-containing agar - *In vitro* co-cultivation assays in liquid and solid media - Preparation and activity testing of crude colicin F_Y_ extracts - *Ex vivo* colicin F_Y_ stability assays in mouse intestinal content - *In vivo* colonization and competition experiments in mouse models with and without streptomycin - Luciferase reporter assays to monitor *in vivo* expression of colicin F_Y_ | [33] |
| *Bifidobacterium adolescentis* DSM 20086 | - Human intestinal epithelial HT-29 cells | - Reduced adherence and invasion of *Y. enterocolitica* to epithelial cells | - Competitive exclusion by blocking adhesion sites and possibly modulating epithelial cell signaling to reduce bacterial invasion | - Co-incubation of *B. adolescentis* with *Y. enterocolitica* on HT-29 cells - Adhesion and invasion assays to quantify bacterial interaction with epithelial cells - Gentamicin protection assay to distinguish intracellular vs. surface-adhered bacteria - Quantification of mRNA expression in intestinal tissue via qRT-PCR - Statistical analysis of CFU and cytokine levels (e.g., IL-8) | [34] |
| *Bifidobacterium adolescentis* Reuter 1963 (ATCC 15705*)* | Not applicable | - Reduced weight loss and *Yersinia* dissemination to the spleen in infected mice - Increased frequency of pDCs and FoxP3⁺ regulatory T cells | - Modulation of the host’s intestinal immune response - Increase in pDCs and Treg cells that suppress inflammation - Maintains epithelial barrier integrity, reducing systemic dissemination of *Y. enterocolitica* | - Oral infection with *Y. enterocolitica* WA-314 (serotype O8) - Feeding with *B. adolescentis* (ATCC 15705) - Streptomycin pre-treatment to facilitate *B. adolescentis* colonization - Use of vancomycin and metronidazole to deplete *B. adolescentis* - Depletion of pDCs via anti-PDCA-1 antibody - CFU quantification from spleen, Peyer’s patches, and feces - Isolation of lamina propria and intraepithelial leukocytes - Flow cytometry analysis of intestinal immune cells (e.g., pDCs, Tregs) - Daily monitoring of weight and sacrifice at 4 days’ post-infection - Statistical analysis using t-tests and ANOVA with post hoc testing | [35] |
| *Lactobacillus plantarum* ATCC^®^ 8014 | Not applicable | - Inhibited *Y. pseudotuberculosis* growth - Stable at low pH, high temperature, and bile salts - No gas production | - Likely production of bacteriocins and other antimicrobial compounds - Confirmed presence of peptides via HPLC-DAD | - Agar diffusion inhibition assays - Supernatant testing via disc and cylinder methods - HPLC-DAD for peptide detection - Antibiotic susceptibility testing (Kirby-Bauer) - Tolerance tests to pH, bile salts, and temperature - Gas production test - Catalase activity assay - Fermentation kinetics in different media (MRS & formulated Pro) - Inoculum preparation and cell density adjustment - Experimental design (randomized block) and statistical analysis (ANOVA, regression) | [36] |
| *Lactococcus lactis* ATCC^®^ 11454 | Not applicable | - Inhibited *Y. pseudotuberculosis* growth - Confirmed production of antimicrobial peptides - Probiotic was bile- and acid-tolerant | - Production of antimicrobial peptides (e.g., VAL-TIR-VAL) identified by HPLC-DAD - Likely bacteriocin-like inhibition | - Co-culture and supernatant-based growth inhibition assays - Antibiotic susceptibility testing (Kirby-Bauer method) - Peptide extraction and identification via HPLC-DAD - Tolerance to low pH and bile salts - Fermentation kinetics (growth, pH, sugar, protein, acid) - Thermal stress viability tests (38°C and 45°C) - Gas and catalase activity assays - Statistical analysis using SAS (PROC MIXED) | [37] |
| *Recombinant Lactococcus lactis* secreting LcrV (experimental colitis model) | Not applicable | - Reduced severity of chemically induced colitis - Increased IL-10 production - Decreased inflammation and tissue damage | - LcrV protein induced anti-inflammatory IL-10 via TLR2 pathway - Downregulated TNF-α, IL-6, and IL-12 expression | - Construction of recombinant L. lactis expressing LcrV or IL-10 - Plasmid construction and molecular cloning - Bacterial strain culture and antibiotic selection - Western blot for LcrV detection - ELISA-based LcrV quantification - Macrophage stimulation assays - TNBS-induced colitis model in mice - DSS-induced colitis model in mice - Histopathological analysis of colon tissues - Cytokine quantification by ELISA - qRT-PCR - Myeloperoxidase activity assay - Use of genetically modified mouse strains - Statistical analysis (Mann–Whitney U test) | [38] |
| *Lactobacillus fermentum* DSMZ 20052 | - HeLa 229 (human epithelial cells) - HT-29 (colonic epithelial cells) | - Inhibited *Y. enterocolitica*-induced IL-8 secretion and inflammatory signaling in epithelial cells | - A secreted glycerophospholipid (<10 kDa) from *L. fermentum* suppresses activation of Rac1, p38 MAPK, and NF-κB pathways triggered by *Y. enterocolitica* invasin, without affecting bacterial adhesion, invasion, or viability | - *Y. enterocolitica* cultured aerobically in LB at 27°C; *L. fermentum* cultured anaerobically in BHI or RPMI 1640 at 37°C - HeLa 229 cells cultured in RPMI 1640 with 10% FCS, glutamine, and antibiotics; antibiotic-free medium used during infection - Coinfection assays using HeLa cells with live or heat-killed bacteria and bacterial supernatants - Supernatants harvested after 40h growth, centrifuged, filter-sterilized, and size-fractionated - Enzymatic treatment of supernatants (proteinase K, phospholipase C, FeCl₃) - Lipid extraction using chloroform/methanol - IL-8 quantification via ELISA - Western blotting for phospho-p38 MAPK - Rac1 GTPase activity assessed by pulldown assay - NF-κB reporter luciferase assay with transient transfection - Statistical analysis using paired Student t-test | [39] |
| *Lactobacillus paracasei* IMPC2.1, *Lactobacillus plantarum* ITM21B and ITM5BG | - Caco-2 (human intestinal epithelial cells) | - Inhibited growth of *Y. enterocolitica* - Nearly complete suppression of urease activity - Improved survival during simulated digestion - Strong adhesion to enterocytes (for IMPC2.1 and ITM5BG) | - Competitive exclusion and antimicrobial effects in co-culture - Reduction in ammonia production via urease inhibition - Not directly due to lactic acid concentration or pH alone | - Co-culture assay with *Y. enterocolitica* and LAB strains - Lactic acid quantification in cocultures - Urease enzyme activity measurement (via ammonia release) - Low pH and bile salt tolerance tests - Simulated gastric and intestinal digestion assays - Adhesion assay using Caco-2 cell monolayers | [40] |
| *Lactobacillus plantarum* C4 (isolated from kefir) | Not applicable | - Shortened colonization of *Yersinia enterocolitica* O9 in Peyer’s patches - Increased TNF-α and IFN-γ production (immune activation) - Elevated intestinal IgA secretion - Did not affect fecal shedding of yersiniae | - Enhances mucosal and systemic immune response (increased of TNF-α, IFN-γ and IgA) - Promotes bacterial clearance from Peyer’s patches - No evidence for direct antibacterial effect or competition (i.e., not via acid or bacteriocins) | - Specific-pathogen-free BALB/c mice treated daily via intragastric gavage with fermented goat milk containing *L. plantarum* C4 (10⁹ CFU) - Control mice received fermented milk or water alone - Oral infection with *Y. enterocolitica* IP383 (serotype O9, biotype 2, pYV⁺) - Enumeration of yersiniae from feces and Peyer’s patches using selective agar - Cytokine (TNF-α, IL-10, IFN-γ) and total IgA levels measured in cecal content and plasma via ELISA - Statistical analysis performed using Student’s t-test and Wilcoxon test (p < 0.05 considered significant) | [41] |
| *Levilactobacillus brevis* 23017 (formerly *Lactobacillus brevis*) | Not applicable | - Reduced colonization of *Y. enterocolitica* in Peyer’s patches and cecum - Attenuated histopathological damage in intestinal tissues - Enhanced antioxidant capacity (increased of SOD, CAT, and GSH; Decreased of MDA) - Improved intestinal integrity (increased of occludin expression) - Increased intestinal IgA (sIgA and anti-*Yersinia* IgA) - Downregulated proinflammatory cytokines (decreased of TNF-α, IL-6, IL-17a, iNOS, and COX-2) - Upregulated anti-inflammatory IL-10 and tight junction proteins - Prevented weight loss and restored normal body weight post-infection | - Enhanced mucosal immunity through increased IgA and regulated cytokine expression - Lowered MDA and increased antioxidant enzymes - Upregulated tight junction protein occluding - Inhibited phosphorylation of p38 MAPK and NF-κB, key inflammatory pathways | - BALB/c mice divided into control, *Y. enterocolitica*, and LAB+*Y. enterocolitica* groups - 21-day oral treatment protocol with NaHCO₃ pre-treatment; 1×10⁹ CFU L. brevis for 7 days followed by *Y. enterocolitica* challenge on day 8 - Mice maintained under controlled housing conditions (20 °C, 50% humidity) with free access to food and water - Histopathological analysis of spleen, liver, and intestinal tissues (H&E staining) - ELISA to measure serum IgG and intestinal sIgA levels - RT-qPCR to evaluate cytokine and barrier-related gene expression - Western blot for phosphorylation of NF-κB and p38 MAPK signaling proteins - Antioxidant assays (MDA, SOD, CAT, GSH) in spleen and intestinal homogenates - Bacterial colony counts in Peyer’s patches and cecal mucosa using CIN agar plates | [42] |
| *Lactobacillus acidophilus* LA-5® | None | - Slowed growth of *Y. enterocolitica* in blue cheese enriched with *L. acidophilus* LA-5^®^ - Reduced bacterial counts at 6 °C, 9 °C, and 12 °C compared to cheese without probiotic - Did not fully prevent pathogen growth or ensure food safety | - Competitive inhibition within the food matrix - Likely production of antimicrobial compounds and/or pH modification - No direct immune modulation (*in vitro* or *in vivo* models not used) | - Cheese inoculated with a mix of *Y. enterocolitica* strains (ATCC 23715, 27729, and a field isolate) - Initial pathogen load: ~10³ CFU/g - Storage at 3 °C, 6 °C, 9 °C, 12 °C, and 15 °C up to 480 hours - CIN agar used for enumeration of *Yersinia* - Comparison between blue cheese with and without L. acidophilus LA-5^®^ - Statistical analysis via ANOVA and Duncan’s test (SPSS) | [43] |

*Note:* ANOVA, Analysis of Variance; ATCC, American Type Culture Collection; BHI, Brain heart infusion; Caco-2, Human epithelial colorectal adenocarcinoma cell line; CAT, Catalase; CFU, Colony-forming unit; CIN agar, Cefsulodin-irgasan-novobiocin agar; COX-2, Cyclooxygenase-2; DSS, Dextran sulfate sodium; DSMZ, Deutsche Sammlung von Mikroorganismen und Zellkulturen (German collection of microorganisms and cell cultures); E. coli, *Escherichia coli*; ELISA, Enzyme-linked immunosorbent assay; FCS, Fetal calf serum; FITC, Fluorescein isothiocyanate; GSH, Glutathione; HeLa, human cervical epithelial cell line derived from Henrietta Lacks; HPLC-DAD, High-performance liquid chromatography with diode-array detection; HT-29, Human colorectal adenocarcinoma cell line; IFN-γ, Interferon gamma; IL, Interleukin; iNOS, Inducible nitric oxide synthase; LcrV, Low calcium response V antigen; LB, Lysogeny broth (Luria-Bertani); MDA, Malondialdehyde; MAPK, Mitogen-activated protein kinase; MLNs, Mesenteric lymph nodes; NaHCO₃, Sodium bicarbonate; NF-κB, Nuclear factor kappa-light-chain-enhancer of activated B cells; pDCs, Plasmacytoid dendritic cells; PBS, Phosphate buffered saline; PFGE, Pulsed-field gel electrophoresis; qRT-PCR, Quantitative reverse transcription polymerase chain reaction; RPMI 1640, Roswell Park Memorial Institute Medium 1640; SOD, Superoxide dismutase; sIgA, Secretory immunoglobulin A; SOE-PCR, Splicing by overlap extension PCR; Tregs, Regulatory T Cells; TNBS, 2,4,6-trinitrobenzenesulfonic acid; TNF-α, Tumor necrosis factor alpha; U937, human monocytic cell line.

**References**

1. D. Aebisher, D. Bartusik-Aebisher, A. Przygórzewska, P. Oleś, P. Woźnicki, and A. Kawczyk-Krupka, “Key Interleukins in Inflammatory Bowel Disease—A Review of Recent Studies,” *International Journal of Molecular Sciences* 26, no. 1 (2024): 121.

2. Z. Cai, S. Wang, and J. Li, “Treatment of Inflammatory Bowel Disease: A Comprehensive Review,” *Frontiers in Medicine* 8 (2021): 765474.

3. J. Lv, Y. S. Ibrahim, A. Yumashev, A. Hjazi, A. Faraz, M. J. Alnajar, et al., “A Comprehensive Immunobiology Review of IBD: With a Specific Glance to Th22 Lymphocytes Development, Biology, Function, and Role in IBD,” *International Immunopharmacology* 137 (2024): 112486.

4. H. Cai, Z. Liu, P. Sun, Y. Zhou, Y. Yan, Y. Luo, et al., “Discovery of a Dual-Acting Inhibitor of Interleukin-1β and STATs for the Treatment of Inflammatory Bowel Disease,” *RSC Medicinal Chemistry* 15, no. 2 (2024): 193–206.

5. E. Shaul, M. A. Conrad, N. Dawany, T. Patel, M. C. Canavan, A. Baccarella, et al., “Canakinumab for the Treatment of Autoinflammatory Very Early Onset Inflammatory Bowel Disease,” *Frontiers in Immunology* 13 (2022): 972114.

6. Y. Cai, X. Jia, L. Xu, H. Chen, S. Xie, and J. Cai, “Interleukin-17 and Inflammatory Bowel Disease: A Two-Sample Mendelian Randomization Study,” *Frontiers in Immunology* 14 (2023): 1238457.

7. M. A. Williams, A. O’Callaghan, and S. C. Corr, “IL-33 and IL-18 in Inflammatory Bowel Disease Etiology and Microbial Interactions,” *Frontiers in Immunology* 10 (2019): 1091.

8. A. Gesiorowski, J. Ettich, J. Werner, C. Wittich, S. Pieper, G. Padrini, et al., “Bispecific Soluble Cytokine Receptor-Nanobody Fusions Inhibit Interleukin (IL-)6 Trans-Signaling and IL-12/23 or Tumor Necrosis Factor (TNF) Signaling,” *Journal of Biological Chemistry* 299 (2023): 105343.

9. U. Wong and R. K. Cross, “Expert Opinion on Interleukin-12/23 and Interleukin-23 Antagonists as Potential Therapeutic Options for the Treatment of Inflammatory Bowel Disease,” *Expert Opinion on Investigational Drugs* 28, no. 5 (2019): 473–479.

10. L. Shakerian, H. Kolahdooz, M. Garousi, V. Keyvani, R. K. Kheder, T. A. Faraj, et al., “IL-33/ST2 Axis in Autoimmune Disease,” *Cytokine* 158 (2022): 156015.

11. A. Toskas, S. Milias, G. Delis, S. Meditskou, A. Sioga, and T. Papamitsou, “Expression of IL-21 and IL-33 in Intestinal Mucosa of Inflammatory Bowel Disease: An Immunohistochemical Study,” *Diagnostics* 13, no. 13 (2023): 2185.

12. Y. E. Hernandez Santana, N. Irwin, and P. T. Walsh, “IL-36: A Therapeutic Target for Ulcerative Colitis?” *Expert Opinion on Therapeutic Targets* 26, no. 6 (2022): 507–512.

13. G. Leon, Y. E. Hernandez Santana, N. Irwin, E. Giannoudaki, S. O’Neill, I. Csizmadia, et al., “IL-36 Cytokines Imprint a Colitogenic Phenotype on CD4+ T Helper Cells,” *Mucosal Immunology* 15, no. 3 (2022): 491–503.

14. J. Xiong, Y.-H. Lin, L.-H. Bi, J.-D. Wang, Y. Bai, and S.-D. Liu, “Effects of Interleukin-4 or Interleukin-10 Gene Therapy on Trinitrobenzenesulfonic Acid-Induced Murine Colitis,” *BMC Gastroenterology* 13 (2013): 165.

15. M. Godala, E. Gaszyńska, K. Walczak, and E. Małecka-Wojciesko, “Role of Serum Interleukin-6, Interleukin-1β and Interleukin-10 in Assessment of Disease Activity and Nutritional Status in Patients with Inflammatory Bowel Disease,” *Journal of Clinical Medicine* 12, no. 18 (2023): 5956.

16. L. Dumoutier, J. Louahed, and J.-C. Renauld, “Cloning and Characterization of IL-10-Related T Cell-Derived Inducible Factor (IL-TIF), a Novel Cytokine Structurally Related to IL-10 and Inducible by IL-9,” *Journal of Immunology* 164, no. 4 (2000): 1814–1819.

17. H. Griffin, L. Ceron-Gutierrez, N. Gharahdaghi, S. Ebrahimi, S. Davies, P. S. Loo, et al., “Neutralizing Autoantibodies against Interleukin-10 in Inflammatory Bowel Disease,” *New England Journal of Medicine* 391, no. 5 (2024): 434–441.

18. M.-C. Li, “IL-10 and Its Related Cytokines for Treatment of Inflammatory Bowel Disease,” *World Journal of Gastroenterology* 10, no. 5 (2004): 620–625.

19. D. F. Zegarra Ruiz, D. V. Kim, K. Norwood, F. B. Saldana-Morales, M. Kim, C. Ng, et al., “Microbiota Manipulation to Increase Macrophage IL-10 Improves Colitis and Limits Colitis-Associated Colorectal Cancer,” *Gut Microbes* 14, no. 1 (2022): 2119054.

20. K. Y. Fung, C. Louis, R. D. Metcalfe, C. C. Kosasih, I. P. Wicks, M. D. W. Griffin, et al., “Emerging Roles for IL-11 in Inflammatory Diseases,” *Cytokine* 149 (2022): 155750.

21. P. Giuffrida, F. Caprioli, F. Facciotti, and A. Di Sabatino, “The Role of Interleukin-13 in Chronic Inflammatory Intestinal Disorders,” *Autoimmunity Reviews* 18, no. 5 (2019): 549–555.

22. T. Breugelmans, W. Arras, B. Oosterlinck, A. Jauregui-Amezaga, M. Somers, B. Cuypers, et al., “IL-22-Activated MUC13 Impacts on Colonic Barrier Function through JAK1/STAT3, SNAI1/ZEB1 and ROCK2/MAPK Signaling,” *Cells* 12, no. 9 (2023): 1224.

23. J. Chen, J. Huang, J. Shi, M. Li, E. Zhao, G. Li, et al., “Nestin+ Peyer’s Patch Resident MSCs Enhance Healing of Inflammatory Bowel Disease through IL-22-Mediated Intestinal Epithelial Repair,” *Cell Proliferation* 56, no. 2 (2023): e13363.

24. A. Fantou, E. Lagrue, T. Laurent, L. Delbos, S. Blandin, A. Jarry, et al., “IL-22BP Production Is Heterogeneously Distributed in Crohn’s Disease,” *Frontiers in Immunology* 13 (2022): 1034570.

25. M. Kuchař, K. Sloupenská, L. Rašková Kafková, Y. Groza, J. Škarda, P. Kosztyu, et al., “Human IL-22 Receptor-Targeted Small Protein Antagonist Suppresses Murine DSS-Induced Colitis,” *Cell Communication and Signaling* 22 (2024): 469.

26. Q. Zhu, D. Korenfeld, A. Suarez-Fueyo, S. Graham, L. Jin, S. Punit, et al., “Epithelial Dysfunction Is Prevented by IL-22 Treatment in a *Citrobacter rodentium*-Induced Colitis Model That Shares Similarities with Inflammatory Bowel Disease,” *Mucosal Immunology* 15, no. 6 (2022): 1338–1349.

27. J. Wang, G. Kang, H. Lu, A. De Marco, H. Yuan, Z. Feng, et al., “Novel Bispecific Nanobody Mitigates Experimental Intestinal Inflammation in Mice by Targeting TNF-α and IL-23p19 Bioactivities,” *Clinical and Translational Medicine* 14, no. 8 (2024): e1636.

28. C. Y. Liu, S. S. Tam, Y. Huang, P. E. Dubé, R. Alhosh, N. Girish, et al., “TNF Receptor 1 Promotes Early-Life Immunity and Protects against Colitis in Mice,” *Cell Reports* 33, no. 5 (2020): 108275.

29. E. Mizoguchi, A. Mizoguchi, H. Takedatsu, E. Cario, Y. P. De Jong, C. J. Ooi, et al., “Role of Tumor Necrosis Factor Receptor 2 (TNFR2) in Colonic Epithelial Hyperplasia and Chronic Intestinal Inflammation in Mice,” *Gastroenterology* 122, no. 1 (2002): 134–144.

30. Z. Etwebi, J. R. Goldsmith, M. Bou-Dargham, Y. Tian, R. Hood, N. Spitofsky, et al., “TIPE2 Promotes Tumor Initiation but Inhibits Tumor Progression in Murine Colitis-Associated Colon Cancer,” *Inflammatory Bowel Diseases* 28, no. 5 (2022): 764–774.

31. Y. Lou, H. Sun, S. Morrissey, T. Porturas, S. Liu, X. Hua, et al., “Critical Roles of TIPE2 Protein in Murine Experimental Colitis,” *Journal of Immunology* 193, no. 3 (2014): 1064–1070.

32. F. Meng, X. Jiang, X. Wang, Q. Zheng, X.-N. Wang, C. Mei, et al., “Tumor Necrosis Factor-Like Cytokine 1A Plays a Role in Inflammatory Bowel Disease Pathogenesis,” *Proceedings of the National Academy of Sciences of the United States of America* 120, no. 3 (2023): e2120771120.

33. J. Bosák, L. Micenková, M. Hrala, K. Pomorská, M. Kunova Bosakova, P. Krejci, et al., “Colicin FY Inhibits Pathogenic *Yersinia enterocolitica* in Mice,” *Scientific Reports* 8 (2018): 12242.

34. J. S. Frick, K. Fink, F. Kahl, M. J. Niemiec, M. Quitadamo, K. Schenk, et al., “Identification of Commensal Bacterial Strains That Modulate *Yersinia enterocolitica* and Dextran Sodium Sulfate-Induced Inflammatory Responses: Implications for the Development of Probiotics,” *Infection and Immunity* 75, no. 7 (2007): 3490–3497.

35. A. Wittmann, I. B. Autenrieth, and J.-S. Frick, “Plasmacytoid Dendritic Cells Are Crucial in *Bifidobacterium adolescentis*-Mediated Inhibition of *Yersinia enterocolitica* Infection,” *PLoS ONE* 8, no. 8 (2013): e71338.

36. H. Jurado-Gámez, B. J. A. Martínez, T. A. Chaspuengal, and Y. F. Y. Calpa, “Evaluación *in vitro* de la Acción de *Lactobacillus plantarum* con Características Probióticas sobre *Yersinia pseudotuberculosis*,” *Biotecnología en el Sector Agropecuario y Agroindustrial* 12, no. 2 (2014): 49–59.

37. H. Jurado Gámez, A. Romero Benavides, and A. Morillo Garces, “Cinética, Prueba de Crecimiento y Efecto de Inhibición de *Lactococcus lactis* sobre *Yersinia pseudotuberculosis*,” *Biotecnología en el Sector Agropecuario y Agroindustrial* 14, no. 1 (2016): 18–28.

38. B. Foligne, R. Dessein, M. Marceau, S. Poiret, M. Chamaillard, B. Pot, et al., “Prevention and Treatment of Colitis with *Lactococcus lactis* Secreting the Immunomodulatory *Yersinia* LcrV Protein,” *Gastroenterology* 133, no. 3 (2007): 862–874.

39. J.-S. Frick, K. Schenk, M. Quitadamo, F. Kahl, M. Köberle, E. Bohn, et al., “*Lactobacillus fermentum* Attenuates the Proinflammatory Effect of *Yersinia enterocolitica* on Human Epithelial Cells,” *Inflammatory Bowel Diseases* 13, no. 1 (2007): 83–90.

40. P. Lavermicocca, F. Valerio, S. L. Lonigro, A. Di Leo, and A. Visconti, “Antagonistic Activity of Potential Probiotic *Lactobacilli* Against the Ureolytic Pathogen *Yersinia enterocolitica*,” *Current Microbiology* 56, no. 2 (2008): 175–181.

41. S. De Montijo-Prieto, E. Moreno, T. Bergillos-Meca, A. Lasserrot, M.-D. Ruiz-López, A. Ruiz-Bravo, et al., “A *Lactobacillus plantarum* Strain Isolated from Kefir Protects against Intestinal Infection with *Yersinia enterocolitica* O:9 and Modulates Immunity in Mice,” *Research in Microbiology* 166, no. 8 (2015): 626–632.

42. Z. Shi, N. Guan, W. Sun, T. Sun, L. Niu, J. Li, et al., “Protective Effect of *Levilactobacillus brevis* Against *Yersinia enterocolitica* Infection in a Mouse Model via Regulating the MAPK and NF-κB Pathway,” *Probiotics and Antimicrobial Proteins* 14, no. 5 (2022): 830–844.

43. A. Zadernowska, W. Chajęcka-Wierzchowska, and M. P. Ogryzek, “Growth Potential of *Yersinia enterocolitica* in Blue Cheese and Blue Cheese with Probiotic *Lactobacillus acidophilus* LA-5®,” *Journal of Food Science and Technology* 52, no. 11 (2015): 7540–7544.
